# Blood Features Associated with Viral Infection Severity: An Experience from COVID-19-Pandemic Patients Hospitalized in the Center of Iran, Yazd

**DOI:** 10.1155/2024/7484645

**Published:** 2024-03-12

**Authors:** Fatemeh Sadeghi-Nodoushan, Mohamad Reza Zare-Khormizi, Seyedhossein Hekmatimoghaddam, Fatemeh Pourrajab

**Affiliations:** ^1^Department of Nutrition, School of Public Health, Shahid Sadoughi University of Medical Sciences, Yazd, Iran; ^2^School of Medicine, Shahid Sadoughi University of Medical Sciences, Yazd, Iran; ^3^Cardiovascular Research Center, Institute of Basic and Clinical Physiology Sciences, Kerman University of Medical Sciences, Kerman, Iran; ^4^Department of Laboratory Sciences, School of Paramedicine, Shahid Sadoughi University of Medical Sciences, Yazd, Iran; ^5^Reproductive Immunology Research Center, Shahid Sadoughi University of Medical Sciences, Yazd, Iran

## Abstract

Pandemics such as coronavirus disease 2019 (COVID-19) can manifest as systemic infections that affect multiple organs and show laboratory manifestations. We aimed to analyze laboratory findings to understand possible mechanisms of organ dysfunction and risk stratification of hospitalized patients in these epidemics. *Methods*. This retrospective study was conducted among patients admitted to COVID-19 referral treatment center, Shahid Sadoughi Hospital, Yazd, Iran, from April 21 to November 21, 2021. It was the fifth peak of COVID-19 in Iran, and Delta (VOC-21APR-02; B.1-617.2) was the dominant and most concerning strain. All cases were positive for COVID-19 by RT-PCR test. Lab information of included patients and association of sex, age, and outcome were analyzed, on admission. *Results*. A total of 466 COVID-19 patients were included in the study, the majority of whom were women (68.9%). The average age of hospitalized patients in male and female patients was 57.68 and 41.32 years, respectively (*p* < 0.01). During hospitalization, abnormality in hematological and biochemical parameters was significant and was associated with the outcome of death in patients. There was incidence of lymphopenia, neutrophilia, anemia, and thrombocytopenia. The changes in neutrophil/lymphocyte (N/L) and hematocrit/albumin (Het/Alb) ratio and potassium and calcium levels were significant. *Conclusion*. Based on these results, new biochemical and hematological parameters can be used to predict the spread of infection and the underlying molecular mechanism. Viral infection may spread through blood cells and the immune system.

## 1. Introduction

The signs and symptoms of many viral diseases, including SARS-CoV-2, acute influenza (H1N1/H5N1/H7N9), dengue fever, and human acquired immunodeficiency virus (HIV) infection, are clinically very similar. However, reports about the underlying mechanism of organ injuries in these viral infections are still limited [1–3]. It is unclear why severe illness and death occur only in a small subset of viral infections that primarily target the lungs of patients. However, in severe cases, it can damage other organs [2–4].

Laboratory changes in severe cases of flu and COVID-19 can be accompanied by acute respiratory syndrome (ARDS) and failure of vital organs such as the liver, kidney, and heart [5–7].

There is always the risk of the emergence of an infectious strain of a virus such as acute influenza (H1N1/H5N1/H7N9) and coronavirus disease 2019 (COVID-19) that can appear as a systemic infection and affect multiple organs and lead to critically ill patients.

Laboratory findings are manifestations that show organ damage and dysfunction and help diagnose the severity of the disease and realize critically ill patients [2, 3].

This study attempted to analyze and classify the laboratory findings of severe COVID-19 infection in order to find new markers or indicators that can rapidly identify critically ill patients as well as to understand the molecular mechanism involved in severe infection and help in the disease treatment.

Previously, some laboratory findings have been reported from COVID-19 cases; however, there are always new indicators that are better predictors of body dysfunction and ARDS [8–10].

This work tried to collect recorded data from patients hospitalized for COVID-19 and analyze them to finding and explaining new hematological/biochemical indicators related to vital organ function such as the kidney, liver, and heart where they are important for diagnosing/predicting COVID-19 courses.

Here, to provide further insight into the pathogenesis of viral infections and their severities, we analyzed the laboratory findings of hospitalized patients with COVID-19. According to the reports in the literature and the parameters evaluated in the study, we tried to find a correlation between laboratory findings and clinical complications (such as nephropathy and cardiovascular risk) caused by acute viruses such as SARS-CoV-2 [8–10].

## 2. Methodology

### 2.1. Study Population and Design

The institutional review board of Payam Noor University of Taft, Yazd, and Head of Shahid Sadoughi Hospital, Yazd, Iran, approved this retrospective and Accountability Act-compliant review of existing medical records and waived the requirement for informed consent.

Collected data were from April 21 to November 21, 2021, at COVID-19 referral treatment center, Shahid Sadoughi Hospital, Yazd, Iran, from biochemical and hematological parameters assessed among admitted COVID-19 patients. It was the fifth peak of COVID-19 in Iran, and Delta (VOC-21APR-02; B.1-617.2) was the dominant and most concerning strain. Hospitalized patients who had RT-PCR positive test for COVID-19 and D-dimer test were included. All admitted patients met the following inclusion criteria mentioned by the Iranian national COVID-19-headquarter instructions and guidelines for COVID-19, Ministry of Heath, and the World Health Organization (WHO) criteria for COVID-19 [11, 12]. Ultimately, 466 COVID-19 hospitalized patients were selected and included.

### 2.2. Data Collection and Sampling

Data were extracted from electronic records of COVID-19 patients refereed to COVID-19 referral treatment center from April 21 to November 21, 2021, and included demographic, clinical, and laboratory data. Blood samples had been collected from patients after admission for analysis of biochemical and hematological parameters, while diet restriction was monitored for 6 hours before sampling. The laboratory records included creatinine (Cr) and urea levels, liver function tests, D-dimer, ferritin, C-reactive protein (CRP), and creatine kinase (CPK) levels, platelet counts, prothrombin time (PT) and activated partial thromboplastin time, hemoglobin (Hb) and complete blood count (CBC), white blood cell (WBC) concentration, red blood cell (RBC) and platelet count (PLT) tests, and electrolyte parameters (Ca^2+^, Na^+^, CI^−^, K^+^, and HCO_3_). Neutrophil/lymphocyte (N/L) and hematocrit/albumin (Het/Alb) ratios were calculated as additional hematological parameters which may be used for fast diagnosis of severe infections [8, 9, 13].

### 2.3. Data Extraction and Statistical Analysis

Electronic records of COVID-19 patients on admission were collected which included laboratory tests and demographic data during routine examination of patients at the hospital. We applied IBM SPSS software (version 22.0) for all statistical analyses. Continuous variables were tested for normality and are reported as mean ± standard deviation or median.

We assessed the correlations among different blood parameters using correlation analysis tests (Pearson's/spearman coefficient, each was appropriate). Binary logistic regression model was used to estimate which biochemical and hematological abnormality can be used as an indicator of the occurrence of death in patients: biochemical cutoffs as an independent variable and the outcome of death as a dependent variable. *p* value <0.05 was considered statistically significant.

## 3. Results

### 3.1. Demographics of Admitted Patients

In this study, clinical and laboratory data of electronic records of 466 hospitalized COVID-19 patients refereed to COVID-19 referral treatment center from April 21 to November 21, 2021, were included. Overall, 31% of admitted patients were male of whom 51% died (Tables 1(a)–1(c)).

Differences in gender and age have an impact on patients' outcome, and male and old patients were, respectively, 2.99 and 2.62 times more at risk of death compared to patients without this condition (Table 1(a)).

Descriptive statistics for all COVID-19-hospitalized patients included in this study are provided in Tables S1–S5 in the supplemental data. Based on the outcomes of COVID-19-included subjects, 66.31% and 33.69% of patients recovered and did not recover in hospital (death outcome), respectively (Tables 1(a) and S3–S5).

**Figure 1:**
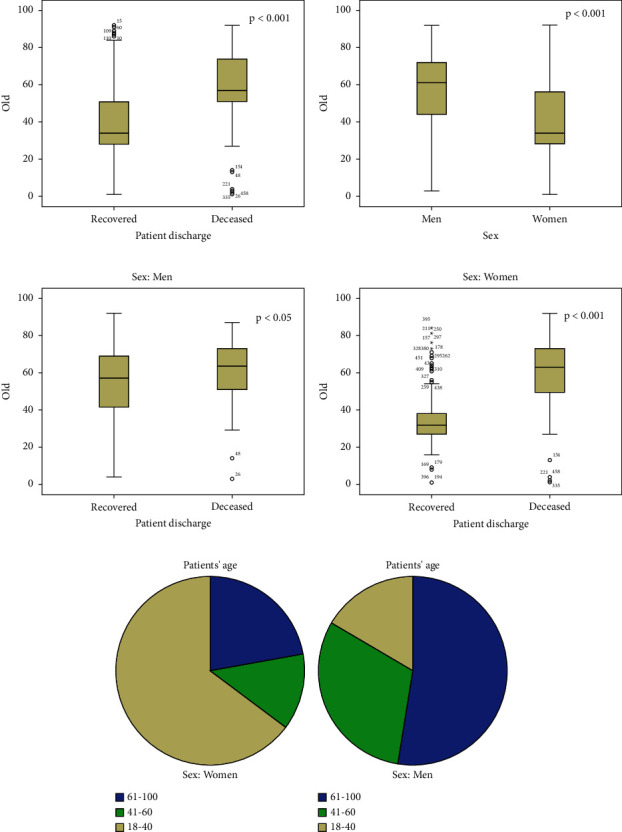
Sex and age characteristics of admitted COVID-19 patients and outcomes, at COVID-19 referral treatment center, Shahid Sadoughi Hospital, Yazd, Iran (women, *n* = 309; men, *n* = 157). (a) The average age of recovered and nonrecovered patients (deceased group) was 39.8 and 59.43 years, respectively. (b) The average age of male and female patients was 57.68 and 41.32 years, respectively. (c, d) The average age of the deceased group in males and females was 60.84 and 58.17 years, respectively. (e) In the age range of female participants, 64.50% of them were between 18 and 40 years old. (f) In the age range of male participants, 12.2% were under 40 years old, and 87.7% were over 40 years old.

The average age of recovered patients and nonrecovered patients (deceased group) was 39.8 and 59.43 years, respectively (*p* < 0.001, Figure 1(a)). The average age of male and female patients was 57.68 and 41.32 years, respectively (*p* < 0.001, Figure 1(b)). The average age of recovered male and female patients was 55 and 35 years, respectively (*p* < 0.05, Figures 1(c) and 1(d)).

Among the hospitalized patients, 145 (31%) were male and 321 (68.9%) were female. In terms of disease severity, 51% of men and 25.9% of women died in hospital (*p* < 0.001, Table 1(a)). Regarding the age range of participants, 64.50% of female patients were between 18 and 40 years old and 35.50% of them were over 40 years of age (*p* ~ <0.000, Figure 1(e)), while 12.2% of male participants were under 40 years of age (*p* ~ 0.05) and the majority of them were over 40 years of age (87.7%, *p* ~ 0.051 (Figure 1(f)).

### 3.2. Blood Groups and Severity of COVID-19

Frequency of blood groups among included patients was different but not found statistically significant compared to the normal frequency in the society. The percentage of patients with blood group B (±) was higher than the normal percentage in the population (*p* ~ 0.5, Table S1A). Binary logistic regression analysis showed the highest odds of death for AB blood group among COVID-19 patients (Table 1(c)).

### 3.3. Biochemical Analysis Results of COVID-19 Patients

Biochemical abnormalities were more frequent in aged than the young and in the male than the female COVID-19 patients (Figure 1). Patients who died in hospital showed higher levels of fasting blood sugar (FBS), lactate dehydrogenase (LDH), glutamate-pyruvate aminotransferase (GOT)/glutamate-oxaloacetate aminotransferase (GPT), alkaline-P (ALP), bilirubin D, urea, creatinine (Cr), sodium (Na^+^), and potassium (K^+^) than recovered patients. In contrast, albumin and calcium (Ca^2+^) levels in deceased patients were lower than normal (*p* < 0.01, Tables S2–S6).

Diabetes mellitus of admitted patients was significantly higher than the normal frequency in the society according to reference [14] and more common in female patients (*p* < 0.001, Table S1B). Diabetic patients were 2.574 times more at risk of progression to the deceased outcome compared to the patients without this disorder (Table 1(a)).

### 3.4. Some Laboratory Findings Related to Liver Function

On admission, 55.4% and 23.2% of patients had serum GOT (SGOT) levels >40 and serum GPT (SGPT) levels >56, respectively (*p* < 0.01) (Table 1(a)). Among patients, 56.6% of males and 39.2% of females had SGOT levels >40 (*p* ~ 0.001) (Table S7). Subjects with SGOT level >40 showed high levels of biochemical markers related to liver dysfunction or muscle injuries: LDH/SGPT/bilirubin D/alkaline-P/CPK (*p* < 0.01, Table S8). Patients with SGOT >40 also exhibited high levels of hematological markers PT, ferritin, neutrophils, and N/L ratio (*p* < 0.05, Table S8).

Patients with SGOT >40 were 2.174 times more at risk to progress to death than patients with SGOT ≤40 (*p* < 0.01, Table 1(a)).

Patients who progressed to death showed significantly higher bilirubin D, alkaline-P, and ferritin levels than those who recovered (*p* < 0.05, Table S6). In patients with deceased outcome, bilirubin D levels showed a significant correlation with alkaline-P, PT, and PLT (*p* < 0.01, Table S18). Generally, bilirubin D >0.2 and alkaline-P >128 caused 7.347 and 1.518 times more risk of progression to the deceased outcome in COVID-19 patients, respectively (Tables 1(a) and S19A).

### 3.5. Some Laboratory Findings Related to Muscles or Heart Function

On admission, 47.9% of patients had CPK levels >200 of whom 34.5% progressed to death (*p* < 0.01) (Table 1(a)). Patients with CPK >200 had significantly higher levels of PT/SGPT/SGOT/urea/creatinine (*p* < 0.01, Tables S9 and S10). In brief, CPK showed a positive correlation with LDH/SGOT/SGPT/urea/creatinine levels (*p* < 0.01, Table S18).

### 3.6. Some Laboratory Findings Related to Kidney Function

Frequency of creatinine levels >1.4 was 13.1% in COVID-19 patients of whom 73.8% died in the hospital (*p* < 0.001, Table 1(a)). Male patients were 4.696 times more likely to suffer from creatinine>1.4 than women (*p* < 0.001, Table S11).

Patients with creatinine levels >1.4 had higher levels of PT/ferritin/Na^+^/K^+^/Pi/Mg^2+^ than those with creatinine ≤1.4 (*p* < 0.01, Table S12A).

Subjects with creatinine >1.4 showed lower levels of Ca^2+^, lymphocytes and PLT than those with creatinine <1.4 (*p* < 0.001, Table S12A).

Regarding urea levels, about 79.3% of males and 53.3% of females had urea levels >40 (*p* < 0.001, Table S11). The probability of death in patients with abnormal levels of urea (>40) and creatinine (>1.4) was 3.410 and 7.358 times higher than those with low urea (≤40) and creatinine (≤1.4) levels, respectively (*p* < 0.001, Tables 1(a) and S19A).

### 3.7. Some Laboratory Findings Related to Serum Major Ions

The frequency of hypocalcemia (Ca^2+^ <8.4) was 41.4%, of whom 46.1% died in the hospital (*p* < 0.001, Table 1(a)). About 50.3% of men and 37.4% of women had calcium levels less than 8.4 (*p* ~ 0.006, Table S11).

Hyperkalemia frequency (K^+^ >5) was 10.9%, of whom 78.4% died in the hospital (*p* < 0.001, Table 1(a)). Male patients showed 4.151 times more risk to develop hyperkalemia (*p* < 0.001, Table S11).

Patients with hyperkalemia and hypocalcemia were 9.231 and 2.567 times more susceptible to progress to the deceased outcome, respectively (*p* < 0.001, Tables 1(a) and S19A).

### 3.8. Hematological Abnormalities in COVID-19 Patients

Hematological characteristics of the patients on admission are presented in Tables S4 and S13. Patients who died in hospital showed significantly higher levels of WBC/neutrophils/CRP/ferritin and PT than recovered patients (*p* < 0.01, Table S13). Compared with the recovered group with N/L and Het/Alb less than 8 and 9, the deceased group had N/L and Het/Alb more than 8 and 9, respectively (*p* < 0.01, Table S13).

Correlation analysis showed that neutrophil counts may be positively correlated with ferritin levels and negatively correlated with PT and PLT (*p* < 0.01, Tables 2 and S18).

The overall magnitude of lymphopenia (<1) was 52.4% of whom 47.1% had deceased outcome (*p* < 0.001, Table 1(b)). About 76.6% of males and 41.1% of females had lymphocytes <1 (*p* < 0.001, Table S14).

Lymphopenia patients had higher levels of N/L and Het/Alb ratios (Table S15). In patients with deceased outcome, lymphocyte correlated negatively with N/L ratio (*p* < 0.01, Tables 2 and S10).

The overall severity of neutrophilia (>8) was 38.2%, of whom 47.8% died in the hospital (*p* < 0.001, Table 1(b)). About 53.1% of male and 31.5% of female patients had neutrophils >8 (*p* < 0.001, Table S14). Patients with neutrophilia or lymphocytopenia showed 3.821 and 6.659 times more risk of progression to death outcome, respectively (*p* < 0.001, Tables 1(b) and S19C).

The frequency of thrombocytopenia (<140) was 28.3% of whom 52.3% progressed to death (*p* < 0.001, Table 1(b)). Male patients were 3.173 times more prone to develop thrombocytopenia than the females. About 45.5% of males and 20.6% of females had thrombocytopenia (*p* < 0.001, Table S14).

The frequency of anemia (<3.5) was 12.5%, of whom 49.2% had deceased outcome (*p* ~ 0.006, Table 1(b)). Male patients were 3.083 times more prone to develop anemia than the females. About 22.1% of males and 8.4% of females showed anemia (*p* < 0.001, Table S14).

Correlation analysis showed that RBC count may be positively correlated with Het/Alb ratio and Ca^2+^ levels (*p* < 0.01, Tables 2, S10, and S18).

The frequency of low hemoglobin levels was almost similar in male and female patients. Patients with hemoglobin <12 showed low levels of bilirubin T, hematocrit, MCH, and MCHC (*p* ~ 0.782, Table S16).

### 3.9. Inflammatory Markers and Severity of COVID-19

Patients with high levels of CRP (1–3), ferritin (more than 300), and PT (more than 200) showed 1.337, 10.981, and 4.463 times more risk of mortality, respectively (*p* < 0.01, Tables 1(c) and S19).

In addition, COVID-19 patients with N/L index >9 and Het/Alb index >10 may be 5.59 and 1.295 times more prone to the progression to the death outcome, respectively (*p* < 0.01, Tables 1(c) and S19).

## 4. Discussion

Pandemics such as COVID-19 and acute influenza (H1N1/H5N1/H7N9) can manifest as systemic infections that affect multiple organs and show laboratory manifestations. There is always need for new biochemical and hematological indicators which can be used to predict the outcome of infection and the underlying molecular mechanism [2, 3, 9].

This retrospective study was conducted to analyze laboratory findings of COVID-19 severe infection to understand possible mechanisms of organ dysfunction and risk stratification of hospitalized patients in these epidemics.

In the case of COVID-19, it has been proposed that SARS-CoV-2 may primarily affect tissues and organs expressing angiotensin-converting enzyme (ACE2), such as the lung, liver, heart, and gastrointestinal tract [9, 15, 16].

In this study, the prevalence of diabetes was high in hospitalized patients, the majority of whom was females (Table S1).

Low levels of albumin and Ca^2+^ and increased levels of ferritin, LDH, CPK, SGOT, SGPT, bilirubin D, alkaline-P, urea, creatinine, Na^+^, and K^+^ were observed in patients (Tables S6–S8). The increase in CPK levels was significantly related to the increase in creatinine, urea, LDH, SGOT, and SGPT levels. Bilirubin D showed a positive correlation with alkaline-P, PLT, and PT (Table S18). In addition, the risk of death from viral infection was significantly associated with cutoff levels of K^+^, creatinine, bilirubin D, urea, and Ca^2+^ (odd ratio (OR) >3, Tables 1(a) and S18).

SARS-CoV-2 may directly bind to ACE2-positive cholangiocytes in the liver and cause liver abnormalities and damage [16]. Human pancreatic islet cells, especially insulin-producing *β*-cells, widely express ACE2. ACE2 is also expressed by microvascular pericytes and pancreatic ductal cells, contributing to pancreatic secretion in the adaptive response to diet [17, 18]. We also found elevated levels of CPK without increase in troponin I that can be associated with focal myofibril necrosis and infiltrating of infected macrophages [19, 20].

Moreover, viral infection can causes coagulation abnormalities such as microthrombosis, diffuse intravascular coagulation (DIC), deep vein thrombosis, and systemic microangiopathy [7, 21, 22]. Organ injuries in severe cases of viral infection have been associated with widespread microthrombosis [4, 5, 21] and organ infiltration of lymphocytes and neutrophils [16–18].

The prevalence of lymphopenia, neutrophilia, thrombocytopenia, and anemia was high in COVID-19-hospitalized patients, the majority of whom progressed to death (Tables 1(b), S11, and S14).

In the alveolar region, macrophages, epithelial cells, and even endothelial cells express ACE2, and their activation through binding of ACE2 to the ligand leads to the release of proinflammatory cytokines, interleukin (IL)-1*β* and IL-18, and in short, the pathogenic Inflammatory responses. In addition, the virus and its particles are recognized by other receptors expressed on professional antigen-presenting cells (APCs), mainly dendritic cells and macrophages. Through these receptors, the virus enters APCs and may be transferred to CD4+ T cells via MHC II presenters [23, 24], thereby inducing IL-6 overexpression and lymphocyte apoptosis [5]. Overactivated macrophages may cause a hyper-inflammatory life-threatening syndrome, associated with high levels of ferritin and CRP in the serum. Overactivated macrophages phagocytose red blood cells, leading to severe peripheral blood cytopenia, a common feature seen in the COVID-19 syndrome [25]. The cytokine storm and uncontrolled inflammation are possible factors for neutrophilia and thrombocytopenia, which are associated with capillary and acute thrombosis [1, 5, 7].

The virus may infect lymphocytes or hematopoietic stem cells (HSCs) in the bone marrow directly because they may express spike receptor (e.g., ACE2) on their surface [7, 26, 27]. Influenza and HIV agents to spread in the host body use a similar mechanism. HIV can infect HSCs as they express receptors of the virus. Abnormalities in ferritin and cytokine release and blood cell counts may be at least partially due to this mechanism [7, 9, 26]. Erythrocytes express surface receptors that may act as attachment sites for infectious agents (such as HIV-1) to attack and kill cells [18, 22, 26]. Specific antigens on the surface of red blood cells can cause the direct or indirect transfer of the virus to the target cells and the spread of the virus in the body and cause susceptibility to the disease [28, 29].

Finally, we found a significant association between gender, age, and diabetes with the increased risk of mortality in hospitalized patients with COVID-19, which may be related to the easy spread of the virus in the body, dysfunction of the cellular immune system, and long-term inflammation in the elderly patients [15, 20, 26].

There are some suggestions for new biomarkers to accurately diagnose the disease and predict organ dysfunction. For example, high serum levels of proinflammatory cytokines (e.g., tumor necrosis factor-*α* (TNF-*α*), IL-6, and interferon-*γ* (IFN-*γ*)) and their associations with miRNA biomarkers may be useful as a prognostic marker in the management and early identification of patients at risk (Table 3) [30, 36, 37, 52, 54].

In pathological conditions, organs or blood cells secrete vesicles enriched in specific miRNAs with important role in clinical and preclinical application [31, 32, 50].

Therefore, monitoring these miRNA patterns (Table 3) along with biochemical and hematological parameters may serve as a prognostic marker in the management and early identification of high-risk patients who require intensive care.

However, there are limitations in the present work since it is a retrospective study on data recorded from biomarkers measured in COVID-19 hospitalized patients.

Data from patients' follow-up after being discharged from the hospital and measuring more molecular biomarkers can enrich the study and help in more reliable interpretation of the results.

## 5. Conclusions and Recommendations

Comprehensive information about viral infections is important for early disease diagnosis, prevention of disease severity, and treatment planning. There are some similarities in the pathogenesis and laboratory manifestations of viral epidemics such as influenza viruses and COVID-19 that may help in understanding the mechanisms of the disease and the severity. Acute influenza (H1N1/H5N1/H7N9) and COVID-19 have appeared as a systemic infection in severe cases that affects multiple organs and shows life-threatening syndrome.

Data analysis and classification of laboratory findings may help to elucidate new markers or indicators for the rapid diagnosis of severe cases of the disease, as well as for a better understanding of the molecular mechanism involved in organ dysfunction or abnormality.

## Figures and Tables

**Table 1:** (a) Descriptive information and biochemical analysis (related to organ function; kidney, heart, and liver) of included patients (aged 18–92 years) admitted to COVID-19 referral treatment center, Shahid Sadoughi Hospital, Yazd, Iran, (*n* = 466), and multivariate analysis of risk factors associated with infection severity and prediction of death outcome. (b) Hematological features of included patients referred to COVID-19 referral center, Shahid Sadoughi Hospital, Yazd, Iran (*n* = 466 people), and multivariate analysis of risk factors that may be related to infection severity and prediction of death outcome. (c) Laboratory indexes of included patients admitted to COVID-19 referral treatment center, Shahid Sadoughi Hospital, Yazd, Iran (*n* = 466), and multivariate analysis of risk factors that may be associated with viral infection severity and death outcome in patients.

(a)								

Variables	Groups	All cases (mean, years)	*p* value	Recovered (mean, years)	Deceased (mean, years)	OR	Multivariate analysis of risk factors for deceased (95% CI)	*p* value

Age (years)	Male	57.68 (18–92)	0.000	54.39 (20–92)	60.84 (18–87)			
	Female	41.32 (18–92)	0.000	35.45 (18–84)	58.17 (18–92)			
	Total	46.41	0.000	39.8	59.43			

	All cases (%)			Recovered (%)	Deceased (%)			

Sex	Male	145 (31)		71 (49)	74 (51)	2.990	1.983–4.50	0.000
	Female	321 (68.9)		238 (74.1)	83 (25.9)			
	Total	466 (100)	0.000	309 (66.31)	157 (33.691)			

Age (years) (men)	18–40	18 (12.2)		13 (72.30)	5 (27.7)			0.267
	41–60	47 (32.4)		24 (51.1)	23 (48.90)	2.625	0.478–14.428	0.04
	61–100	80 (55.3)	0.050	31 (37.75)	49 (62.25)	4.60	0.87–24.316	0.031

Age (years) (women)	18–40	207 (64.5)		191 (92.4)	16 (7.6)			0.804
	41–60	43 (13.4)		25 (59.5)	17 (40.5)	1.190	0.301–4.703	0.068
	61–100	71 (22.1)	0.000	24 (33.8)	47 (66.2)	3.427	0.913–12.871	0.000

Variables	Cutoffs^a^	All cases (%)	*p* value^b^	Recovered (%)	Deceased (%)	OR^c^	Multivariate analysis of risk factors for deceased (95% CI)	*p* value

FBS	<126^*∗*^	56 (10)		23 (46)	27 (54)			
	≥126^*∗∗*^	410 (90)	0.001	285 (68.7)	130 (31.3)	2.574	1.422–4.659	0.002

Creatinine	≤1.4	405 (86.9)		293 (72.3)	112 (27.7)			
	>1.4	61 (13.1)	0.000	16 (26.2)	45 (73.8)	7.358	3.995–13.550	0.000

Urea	≤40	180 (38.6)		147 (81.7)	33 (18.3)			
	>40	286 (61.4)	0.000	162 (56.6)	124 (43.4)	3.41	2.187–5.316	0.000

SGOT	≤40	258 (44.6)		191 (74)	67 (26)			
	>40	208 (55.4)	0.000	118 (56.7)	90 (43)	2.174	1.471–3.214	0.000

SGPT	≤56	358 (76.8)		251 (70.1)	107 (29.9)			
	>56	108 (23.2)	0.002	58 (53.7)	50 (46.3)	2.022	1.301–3.142	0.002

Alkaline-P	≤128	79 (17)		57 (72.2)	22 (27.8)			
	>128	387 (83)	0.228	252 (65.1)	135 (34.9)	1.518	0.842–2.739	0.166

Bilirubin D (mg/dL)	≤0.2	291 (62.4)		206 (70.8)	85 (29.2)			
	>0.2	175 (37.6)	0.008	103 (58.9)	72 (41)	5.605	1.602–19.612	0.007

Hypocalcemia	<8.4	193 (41.4)		104 (53.9)	89 (46.1)	2.567	1.731–3.808	0.000
	≥8.4	272 (58.4)	0.000	204 (75)	68 (25)			

Hyperkalemia	≤5	414 (88.8)		297 (71.7)	117 (28.3)			
	>5	51 (10.9)	0.000	11 (21.6)	40 (78.4)	9.231	4.581–18.602	0.000

CPK (U/L)	≤200	243 (52.1)		163 (67.10)	80 (32.90)			
	>200	223 (47.9)	0.000	146 (65.50)	77 (34.50)	1.075	0.732–1.578	0.714

(b)								

Variables	Cutoffs^d^	All cases (%)	*p* value^e^	Recovered (%)	Deceased (%)	OR^f^	Multivariate analysis of risk factors for deceased (95% CI)	*p* value

Hemoglobin	<12^*∗*^	201 (43.1)		135 (67.20)	66 (32.8)	0.935	0.634–1.379	0.734
	≥12^*∗∗*^	265 (56.9)	0.000	174 (65.70)	91 (34.3)			

Anemia^g^	<3.5	59 (12.5)		30 (50.8)	29 (49.2)	2.107	1.214–3.658	0.008
	≥3.5	407 (87.3)	0.006	279 (68.6)	128 (31.4)			

Neutrophilia^h^	≤8	288 (61.8)		224 (77.8)	64 (22.2)			
	>8	178 (38.2)	0.000	85 (47.8)	93 (52.2)	3.813	2.086–6.972	0.000

Lymphocytopenia^i^	<1	244 (52.4)		129 (52.9)	115 (47.1)	6.659	3.789–11.705	0.001
	≥1	222 (47.6)	0.000	189 (81.1)	42 (18.9)			

Thrombocytopenia^j^	<140	132 (28.3)		63 (47.7)	69 (52.3)	3.062	2.013–4.657	0.000
	≥140	334 (71.7)	0.000	246 (73.7)	88 (26.3)			

PT^k^	≤13	281 (60.3)		215 (76.5)	66 (23.5)			
	>13	185 (39.7)	0.000	94 (50.8)	91 (49.20)	4.463	2.648–7.522	0.000

Blood groups	A−	19 (4.1)		12 (3.9)	7 (4.5)	1.361	0.501–3.699	0.546
	A+	105 (22.5)		70 (22.7)	35 (22.3)	1.167	0.678–2.009	0.578
	AB−	3 (0.6)		2 (0.6)	1 (0.6)	1.167	0.103–13.219	0.901
	AB+	19 (4.1)		10 (3.20)	9 (5.7)	2.100	0.796–5.542	0.134
	B−	18 (3.9)		10 (3.2%)	8 (5.1)	1.867	0.688–5.061	0.220
	B+	139 (29.8)		93 (30.1)	46 (29.3)	1.154	0.696–1.913	0.578
	O−	23 (4.9)		14 (4.5)	9 (5.7)	1.500	0.603–3.734	0.384
	O+	140 (30)		98 (31.7)	42 (26.8)			0.801

(c)								

Variables	Cutoffs^l^	All cases (%)	*p* value^m^	Recovered (%)	Deceased (%)	OR^n^	Multivariate analysis of risk factors for deceased (95% CI)	*p* value

N/L ratio^o^	≤9^*∗*^	187 (40.1)		227 (81.4)	52 (18.6)			
	>9^*∗∗*^	279 (59.9)	0.000	82 (43.9)	105 (56.1)	5.59	3.682–8.485	0.000

D-Dimer (ng/mL)^p^	≤200	206 (44.2)		144 (69.90)	62 (30.10)			
	>200	260 (55.8)	0.144	165 (63.50)	95 (36.50)	1.337	0.905–1.976	0.144

CRP (N/P)^q^	0	50 (10.7)		45 (90)	5 (10)			0.005
	1	56 (12)		37 (66.10)	19 (33.90)	4.622	1.574–13.567	0.000
	2	236 (50.6)		143 (66.60)	93 (39.40)	5.853	2.241–15.288	0.004
	3	124 (26.6)	0.000	84 (67.70)	40 (32.30)	4.714	1.580–11.623	0.003

Ferritin^r^	≤300	87 (18.7)		82 (94.3)	5 (5.7)			
	>300	379 (81.3)	0.000	227 (59.9)	152 (40.1)	10.981	4.35–27.719	0.000

Het/Alb^s^	≤10	277 (59.4)		206 (74.4)	71 (25.6)			
	>10	189 (40.6)	0.000	103 (54.5)	86 (45.5)	2.423	1.635–3.590	0.000

^a^Patient data were divided into two categories: those who died in the hospital and those who were discharged from the hospital, and then the information was analyzed by logistic regression to identify possible potential risk factors. The cutoffs were selected according to the normal range of biochemical markers. ^b^Patients were divided into two categories: those who died in the hospital and those who were discharged from the hospital, and then their frequencies in each group were analyzed and compared. ^c^The logistic regression analysis was done to estimate which indicator can be related to the occurrence of death in patients: biochemical cutoffs as independent variables and the outcome of death as a dependent variable. ^*∗*^≤: the serum ranges equal to or less than the normal reference range. ^*∗∗*^≥: the serum ranges equal to or more than the normal reference range. FBS: fasting blood sugar; SGOT: serum glutamate-pyruvate aminotransferase; GPT: serum glutamate-oxaloacetate aminotransferase; CPK: creatine kinase. ^d^Patients were divided into subgroups based on specific cutoff of each variable that showed associations with the deceased outcome including hemoglobin (cutoff <12), anemia (cutoff <3.5), neutrophilia (cutoff >8), lymphocytopenia (cutoff <1), thrombocytopenia (cutoff <140), prothrombin time (PT) (cutoff <13), and blood groups (A−/A+, AB−/AB+, B−/B+, and O−/O+). ^e^Patients were divided into two categories: those who died in the hospital and those who were discharged from the hospital, and then their frequencies in each group were analyzed and compared. ^f^The logistic regression analysis was done to estimate which indicator can predict the occurrence of death in patients: hematological cutoffs as an independent variable and the outcome of death as a dependent variable. ^g^Cases with RBC count <3.5 × 10^12^/L. ^h^Cases with neutrophil count >8 × 10^3^/*µ*L. ^i^Cases with lymphocyte count <1 × 10^3^/*µ*L. ^j^Cases with platelet count <140 × 10^3^/*µ*L. ^k^Cases with prothrombin time (sec). ^l^Patients were divided into subgroups based on a specific cutoff of each variable that showed associations with the deceased outcome including N/L ratio (cutoff >9), D-dimer (cutoff >200), CRP (cutoff∼ +1, +2, +3), ferritin (cutoff >300), and Het/Alb index (cutoff >10). ^m^Patients were divided into two categories: those who died in the hospital and those who were discharged from the hospital, and then their frequencies in each group were analyzed and compared. ^n^The logistic regression analysis was done to estimate which indicator can be related to the occurrence of death in patients: biomarker cutoff as an independent variable and the outcome of death as a dependent variable. N/L: neutrophil/lymphocyte; CRP: C-reactive protein; Het/Alb: hematocrit/albumin. ^o^Cases with N/L ratio >9; ^p^Cases with D-Dimer (ng/mL) >200; ^q^Cases with CRP(N/P) (+1, +2, +3); ^r^Cases with Ferritin >300; ^s^Het/Alb ratio >10.

**Table 2:** Possible correlations between hematological and biochemical parameters in two groups of COVID-19 patients.

Correlated parameters^a^	Recovered		Deceased	
	*r*	*p* value	*r*	*p* value
Hb-RBC	0.681^*∗∗*^	0.000	0.844^*∗∗*^	0.000
Hb-Het/Alb	0.593^*∗∗*^	0.000	0.550^*∗∗*^	0.000
Hb-Ca	0.167^*∗∗*^	0.003	0.293^*∗∗*^	0.000
RBC-Het/Alb	0.439^*∗∗*^	0.000	0.497^*∗∗*^	0.000
RBC-Ca	0.141^*∗*^	0.013	0.273^*∗∗*^	0.001
PT-PLT	−0.125^*∗*^	0.029	−0.197^*∗*^	0.014
PT-PDW	0.102	0.072	0.201^*∗*^	0.012
Lymphocyte-neutrophil	−0.918^*∗∗*^	0.000	−0.855^*∗∗*^	0.000
Neutrophil-N/L	0.940^*∗∗*^	0.000	0.875^*∗∗*^	0.000
Lymphocyte-N/L	−0.995^*∗∗*^	0.000	−0.997^*∗∗*^	0.000
Lymphocyte-RBC	−0.210^*∗∗*^	0.000	−0.028	0.731
Lymphocyte-Het/Alb	−0.209^*∗∗*^	0.000	−0.15	0.06
Lymphocyte-WBC	−0.437^*∗∗*^	0.000	−0.428^*∗∗*^	0.000
Neutrophil-WBC	0.394^*∗∗*^	0.000	0.434^*∗∗*^	0.000
Lymphocyte-ferritin	−0.163^*∗∗*^	0.004	0.011	0.896
Neutrophil-ferritin	0.193^*∗∗*^	0.000	−0.026	0.743
Alb-Het/Alb	−0.500^*∗∗*^	0.000	−0.546^*∗∗*^	0.000

^a^Patients were divided into two groups: those who died in the hospital (deceased group) and those who were discharged from the hospital (recovered group), and then the possible correlations between hematological and biochemical parameters were evaluated in each group, separately. ^*∗*^Correlation is significant at the 0.05 level (2-tailed). ^*∗∗*^Correlation is significant at the 0.01 level (2-tailed). Hb: hemoglobin; RBC: red blood cell; Alb: albumin; N/L: neutrophil/lymphocyte; Het/Alb: hematocrit/albumin; WBC: white blood cell count; PLT: platelet count; PDW: platelet distribution width; Ca: calcium.

**Table 3:** Circulating biomarkers and associated miRNAs related to organ dysfunction and abnormalities in COVID-19.

Infection-related organ dysfunction	Classic biomarkers	Elevated miRNAs (show significant association with biomarkers)	References
Acute respiratory distress syndrome (ARDS)	D-Dimer, PT, PLT%, IL-1*β*, TNF, IFN-*γ*, IL-6, IL-8, IL-10 [9, 25, 26]	miR-181, miR-101, miR-155, miR-320, miR-92, miR-93, miR-144, miR-34a	[30–35]
Systemic inflammatory response [1, 16, 21]	Ferritin, CRP, N/L, PT, Het/Alb, IL-1*β*, TNF, IFN-*γ*, IL-6, IL-8, IL-10 [9, 25, 26]	miR-223, miR-222, miR-126, miR-155, miR-320, miR-93, miR-142	[33, 36–39]
Neutrophilia	Neutrophil count, WBC, N/L	miR-155, miR-320, miR-93, miR-146a, miR-221, miR-29, miR-21,	[30, 40–43]
Lymphocytopenia	Lymphocyte count, WBC, N/L, ferritin, IL-1*β*, TNF, IFN-*γ*, IL-6 [5, 25]	miR-155, miR-320, miR-93, miR-144, miR-223	[33–36]
Thrombocytopenia	PLT count, PT, PDW, IL-1*β*, TNF, IFN-*γ* [25]	miR-126, miR-320, miR-223, miR-197, miR-191, miR-21, miR-96, miR-98	[42, 44, 45]
Anemia	RBC, Hb, RDW, LDH, K^+^, IL-1*β*, TNF, IFN-*γ* [25]	miR-144, miR-320	[46–49]
WBC count	Leukocyte count, N/L, Het/Alb	miR-150, miR-143, miR-223, miR-155, miR-320, miR-93	[30, 33, 34, 36, 40, 42, 48–51]
Endothelial dysfunction [4, 17, 18]	PLT count, PT	miR-126, miR-223, miR-222, miR-34a	[33, 39]
Coagulation abnormalities (DIC, DVT, and multiorgan thrombosis) [7, 21, 22]	D-Dimer, PT, PLT%	miR-221, miR-222, miR-223, miR-146, miR-126, miR-155, miR-21, miR-320	[31, 40, 42, 44, 45, 52, 53]
Cholangiocyte/bile duct dysfunction and hepatocyte damage [16, 17]	Alkaline-P, LDH, SGOT, SGPT, bilirubin	miR-122 (early), miR-34a, miR-193, miR-187-3p	[54, 55]
Kidney function impairment and injuries	Urea, Cr, Na^+^, K^+^, hypocalcaemia	miR-21, miR-210, miR-34, miR-92a, miR-93, miR-223	[36, 56–59]
Myopathic changes, myofibril necrosis [19, 20]	CPK, LDH, SGOT, SGPT, urea, Cr	miR-133a, miR-155, miR-126, miR-206, miR-208a, miR-34	[52, 60–62]

PLT: platelet; PT: prothrombin time; CRP: C-reactive protein; N/L: neutrophil/lymphocyte; Het/Alb: hematocrit/albumin; LDH: lactate dehydrogenase; SGOT: serum glutamate-pyruvate aminotransferase; GPT: serum glutamate-oxaloacetate aminotransferase; CPK: creatine kinase; WBC: white blood cell; RBC: red blood cell; DIC: disseminated intravascular coagulation; DVT: deep vein thrombosis; Cr: creatinine.

## Data Availability

Datasets used or analyzed during the current study are available from the corresponding author upon reasonable request.

## Additional Points

*Clinical Perspectives*. (i) Viral infections such as acute influenza (H1N1/H5N1/H7N9) and COVID-19 can appear as a systemic infection that affects multiple organs and shows laboratory manifestations. (ii) We analyzed and classified the laboratory findings of viral infection and tried to elucidate the possible organ damage that indicates the severity of the disease. (iii) Understanding the molecular mechanism involved in organ dysfunction and damage in viral infections can help improve the clinical course and proper performance in the treatment of critically ill patients.

## Supplementary Materials

Supplementary Materials Table 1A: analysis of blood groups of COVID-19 hospitalized patients at COVID-19 referral treatment center, Shahid Sadoughi Hospital, Yazd, Iran (*n* = 466). Table 1B: analysis of FBS of COVID-19 hospitalized patients on admission at COVID-19 referral treatment center, Shahid Sadoughi Hospital, Yazd, Iran (*n* = 466). Table 2A: biochemical features of included COVID-19 patients on admission in COVID-19 referral treatment center, Shahid Sadoughi Hospital, Yazd, Iran, (*n* = 466). Table 2B: hematological features of included COVID-19 patients on admission in COVID-19 referral treatment center, Shahid Sadoughi Hospital, Yazd, Iran (*n* = 466). Table 2C: blood electrolyte features of included COVID-19 patients on admission at COVID-19 referral treatment center, Shahid Sadoughi Hospital, Yazd, Iran (*n* = 466). Table 3A: biochemical features of included COVID-19 patients on admission, who recovered on discharge, at COVID-19 referral treatment center, Shahid Sadoughi Hospital, Yazd, Iran, (*n* = 466). Table 3B: biochemical features of included COVID-19 patients on admission, who had death outcome, at COVID-19 referral treatment center, Shahid Sadoughi Hospital, Yazd, Iran (*n* = 466). Table 4A: hematological features of included COVID-19 patients on admission, who recovered on discharge, at COVID-19 referral treatment center, Shahid Sadoughi Hospital, Yazd, Iran (*n* = 466). Table 4B: hematological features of included COVID-19 patients on admission, who had death outcome, at COVID-19 referral treatment center, Shahid Sadoughi Hospital, Yazd, Iran (*n* = 466). Table 5A: blood electrolyte features of included COVID-19 patients on admission, who recovered on their discharge, at COVID-19 referral treatment center, Shahid Sadoughi Hospital, Yazd, Iran (*n* = 466). Table 5B: blood electrolyte features of included COVID-19 patients on admission, who had death outcome, at COVID-19 referral treatment center, Shahid Sadoughi Hospital, Yazd, Iran (*n* = 466). Table 6: comparison of biochemical parameters between two recovered and nonrecovered groups of COVID-19 hospitalized patients, on admission (*n* = 466). Table 7: some laboratory findings of hospitalized patients on admission with SGOT/SGPT higher than the normal (SGOT >40 and SGPT >56) and its association with sex and age (*n* = 466). Table 8: some laboratory findings of hospitalized patients on admission with SGOT/SGPT higher than the normal (SGOT >40 and SGPT >56) and its association with sex and age (*n* = 466). Table 9A: the magnitude of CPK >200 in hospitalized patients, on admission, and its association with sex. Table 9B: some laboratory findings of hospitalized patients, on admission, with different levels of CPK> <200 and its association with sex and age. Table 10: some laboratory findings of hospitalized patients on admission with different serum levels of CPK> ≤200 and its association with deceased outcome. Table 11: the magnitude of some biochemical abnormalities in hospitalized patients, on admission, and its association with sex. Table 12A: some laboratory findings of admitted patients showing serum levels of creatinine >1.4. Table 12B: some laboratory findings of admitted patients showing serum levels of creatinine >1.4, and its association with age. Table 13A: comparison of some hematological parameters between two recovered and nonrecovered groups of COVID-19 hospitalized patients, on admission (*n* = 466). Table 13B: comparison of some hematological parameters between two recovered and nonrecovered groups of COVID-19 hospitalized patients, on admission (*n* = 466). Table 14: the magnitude and laboratory findings of some hematological abnormalities in hospitalized patients on admission and the association with sex. Table 15A: some laboratory findings of admitted patients with lymphocyte counts ≥<1. Table 15B: some laboratory findings of admitted patients with lymphopenia (<1) and its association with deceased outcome. Table 16A: the magnitude and laboratory findings of hemoglobin <12 in hospitalized patients on admission and the association with sex. Table 16B: some laboratory findings of admitted patients with serum levels of hemoglobin ≥<12. Table 17: some laboratory findings of admitted patients with hemoglobin ≥<12 and its association with deceased outcome. Table 18: correlations between biochemical parameters in COVID-19 patients with recovered or deceased outcomes. Table 19A: multivariate analysis of biochemical factors and inflammatory markers that might be associated with COVID-19 risk of death. Table 19B: multivariate analysis of inflammatory markers that may be associated with COVID-19 risk of death. Table 19C: multivariate analysis of hematological factors that may be associated with COVID-19 risk of death. Table 20A: multivariate analysis for the association of age with lymphopenia and neutrophilia in COVID-19 admitted patients (*n* = 466). Table 20B: multivariate analysis for the association of age with lymphopenia and neutrophilia in COVID-19 admitted patients (*n* = 466).
