# Supplementary material for: Blood Features Associated with Viral Infection Severity: An Experience from COVID-19-Pandemic Patients Hospitalized in the Center of Iran, Yazd
Source: Int J Clin Pract. 2024 Mar 12;2024:7484645. doi: 10.1155/2024/7484645 (PMC10950416; doi:10.1155/2024/7484645)
Supplement: Supplementary Materials — Table 1A: analysis of blood groups of COVID-19 hospitalized patients at COVID-19 referral treatment center, Shahid Sadoughi Hospital, Yazd, Iran (n = 466). Table 1B: analysis of FBS of COVID-19 hospitalized patients on admission at COVID-19 referral treatment center, Shahid Sadoughi Hospital, Yazd, Iran (n = 466). Table 2A: biochemical features of included COVID-19 patients on admission in COVID-19 referral treatment center, Shahid Sadoughi Hospital, Yazd, Iran, (n = 466). Table 2B: hematological features of included COVID-19 patients on admission in COVID-19 referral treatment center, Shahid Sadoughi Hospital, Yazd, Iran (n = 466). Table 2C: blood electrolyte features of included COVID-19 patients on admission at COVID-19 referral treatment center, Shahid Sadoughi Hospital, Yazd, Iran (n = 466). Table 3A: biochemical features of included COVID-19 patients on admission, who recovered on discharge, at COVID-19 referral treatment center, Shahid Sadoughi Hospital, Yazd, Iran, (n = 466). Table 3B: biochemical features of included COVID-19 patients on admission, who had death outcome, at COVID-19 referral treatment center, Shahid Sadoughi Hospital, Yazd, Iran (n = 466). Table 4A: hematological features of included COVID-19 patients on admission, who recovered on discharge, at COVID-19 referral treatment center, Shahid Sadoughi Hospital, Yazd, Iran (n = 466). Table 4B: hematological features of included COVID-19 patients on admission, who had death outcome, at COVID-19 referral treatment center, Shahid Sadoughi Hospital, Yazd, Iran (n = 466). Table 5A: blood electrolyte features of included COVID-19 patients on admission, who recovered on their discharge, at COVID-19 referral treatment center, Shahid Sadoughi Hospital, Yazd, Iran (n = 466). Table 5B: blood electrolyte features of included COVID-19 patients on admission, who had death outcome, at COVID-19 referral treatment center, Shahid Sadoughi Hospital, Yazd, Iran (n = 466). Table 6: comparison of biochemical parameter [file 7484645.f1.doc]

**Table 1A.** Analysis of blood groups of COVID-19 hospitalized patients, at COVID-19 referral treatment center, Shahid Sadoughi Hospital, Yazd, Iran (n=466).

|  | ***O+*** | ***A+*** | ***B+*** | ***AB+*** | ***O−*** | ***A−*** | ***B−*** | ***AB−*** | ***p-value*** |
| --- | --- | --- | --- | --- | --- | --- | --- | --- | --- |
| ***Expected Blood group cases (%)*** | 33.5 | 27 | 22.2 | 7 | 4 | 3 | 2.5 | 0.8 |  |
| ***Observed blood group cases (%)*** | 30.26 | 22.75 | 30.04 | 4.29 | 5.15 | 4.29 | 4.08 | 0.86 | 0.461 |

**Table 1B.** Analysis of FBS of COVID-19 hospitalized patients on admission, at COVID-19 referral treatment center, Shahid Sadoughi Hospital, Yazd, Iran (n=466).

|  | ***Diabetes (FBS >126)*** | ***Non-diabetes (FBS< 126)*** | ***p-value*** |
| --- | --- | --- | --- |
| ***Expected cases (n=100)(%)*** | 14.1 | 85.9 |  |
| ***Observed cases (n=466)(%)*** | 419 (90) | 47 (10) | <0.001 |
| ***Recovered cases (n=308)(%)*** | 285 (68.7) | 23 (46) |  |
| ***Deceased cases (n=157)(%)*** | 130 (31.3) | 27 (54) | 0.001 |

**Table 2A.** Biochemical features of included COVID-19 patients on admission, in COVID-19 referral treatment center, Shahid Sadoughi Hospital, Yazd, Iran, (n=466).

|  | ***CPK*** | ***LDH*** | ***SGOT (AST)*** | ***SGPT (ALT)*** | ***Alkaline - P*** | ***Bilirubin D*** | ***Bilirubin T*** | ***Albumin Serum*** | ***Ferritin*** | ***Urea*** | ***Creatinine*** | ***FBS*** |
| --- | --- | --- | --- | --- | --- | --- | --- | --- | --- | --- | --- | --- |
| ***Counts*** | 466.00 | 466.00 | 466.00 | 466.00 | 466.00 | 466.00 | 466.00 | 466.00 | 466.00 | 466.00 | 466.00 | 466.00 |
| ***Average*** | 231.4 | 909.2 | 52.8 | 48.8 | 224.6 | 0.3 | 0.8 | 3.9 | 301.3 | 49.6 | 1.3 | 181.8 |
| ***Percent <^a^*** | 3.2 | 0.0 | 0.0 | 0.2 | 0.0 | 0.0 | 0.0 | 21.0 | 0.7 | 12.8 | 15.7 | 1.9 |
| ***Percent >< ^b^*** | 74.2 | 0.2 | 57.5 | -0.2 | 18.2 | 91.3 | 92.7 | 79.0 | 64.2 | 46.5 | 67.3 | 12.7 |
| ***Percent > ^c^*** | 22.6 | 99.8 | 42.5 | 100.0 | 81.8 | 8.7 | 7.3 | 0.0 | 35.1 | 40.7 | 17.0 | 85.4 |

a: % of included COVID-19 patients showed the biochemical features lower than the normal range on admission.

b: % of included COVID-19 patients showed the biochemical feature between the normal range on admission.

C: % of included COVID-19 patients showed the biochemical features higher than the normal range on admission.

**Table 2B.** Hematological features of included COVID-19 patients on admission, in COVID-19 referral treatment center, Shahid Sadoughi Hospital, Yazd, Iran (n=466)

|  | ***Hematocrit*** | ***Hemoglobin*** | ***MCH*** | ***MCHC*** | ***MCV*** | ***MPV*** | ***PDW*** | ***PLT*** | ***R.B.C*** | ***W.B.C*** | ***PTT*** | ***PT*** | ***ESR_1 hr*** | ***Neutrophil*** | ***Lymphocyte*** | ***N/L*** | ***Het/Alb*** |
| --- | --- | --- | --- | --- | --- | --- | --- | --- | --- | --- | --- | --- | --- | --- | --- | --- | --- |
| ***Counts*** | 466 | 466 | 466 | 466 | 466 | 466 | 466 | 466 | 466 | 466 | 466 | 466 | 466 | 466 | 466 | 466 | 466 |
| ***Average*** | 37.0 | 12.1 | 28.7 | 32.7 | 87.7 | 10.4 | 13.7 | 194.1 | 141.2 | 10.2 | 38.1 | 13.6 | 54.8 | 80.7 | 14.5 | 12.9 | 9.6 |
| ***Percent <^a^*** | 33.5 | 42 | 8.8 | 14.1 | 5.1 | 0.0 | 3.6 | 28.3 | 12.5 | 5.7 | 0.0 | 0.0 | 0.0 | 2.0 | 65.6 | 2.0 | 0.0 |
| ***Percent >< ^b^*** | 66.5 | 58 | 90.3 | 85.2 | 91.9 | 86.6 | 88.2 | 70.6 | 86.8 | 70.5 | 87.3 | 0.5 | 24.9 | 20.7 | 32.5 | 14.9 | 100.0 |
| ***Percent > ^c^*** | 0.0 | 0.6 | 0.9 | 0.7 | 3.1 | 13.4 | 8.1 | 1.1 | 0.7 | 23.7 | 12.7 | 99.5 | 75.1 | 77.3 | 2.0 | 83.1 | 0.0 |

a: % of included COVID-19 patients showed the biochemical features lower than the normal range on admission.

b: % of included COVID-19 patients showed the biochemical feature between the normal range on admission.

C: % of included COVID-19 patients showed the biochemical features higher than the normal range on admission.

**Table 2C.** Blood electrolyte features of included COVID-19 patients on admission, at COVID-19 referral treatment center, Shahid Sadoughi Hospital, Yazd, Iran (n=466).

|  | ***Sodium*** | ***potassium*** | ***Calcium*** | ***Phosphorus*** | ***Magnesium*** | ***ph*** |
| --- | --- | --- | --- | --- | --- | --- |
| ***Counts*** | 466 | 466 | 466 | 466 | 466 | 466 |
| ***Average*** | 136.5 | 4.2 | 8.5 | 3.3 | 2.2 | 7.3 |
| ***Percent <^a^*** | 27.3 | 7.1 | 92.0 | 39.0 | 1.9 | 35.7 |
| ***Percent >< ^b^*** | 70.7 | 83.5 | 7.8 | 53.2 | 59.3 | 39.7 |
| ***Percent > ^c^*** | 2.0 | 9.4 | 0.3 | 7.9 | 38.8 | 24.6 |

a: % of included COVID-19 patients showed the biochemical features lower than the normal range on admission.

b: % of included COVID-19 patients showed the biochemical feature between the normal range on admission.

C: % of included COVID-19 patients showed the biochemical features higher than the normal range on admission.

**Table 3A.** Biochemical features of included COVID-19 patients on admission, who recovered on discharge, COVID-19 referral treatment center, Shahid Sadoughi Hospital, Yazd, Iran, (n=466).

|  | ***CPK*** | ***LDH*** | ***SGOT (AST)*** | ***SGPT (ALT)*** | ***Alkaline - P*** | ***Bilirubin D*** | ***Bilirubin T*** | ***Serum Albumin*** | ***Ferritin*** | ***Urea*** | ***Creatinine*** | ***FBS*** |
| --- | --- | --- | --- | --- | --- | --- | --- | --- | --- | --- | --- | --- |
| ***Counts*** | 309 | 309 | 309 | 309 | 309 | 309 | 309 | 309 | 309 | 309 | 309 | 309 |
| ***Average*** | 222.19 | 684.04 | 43.06 | 40.66 | 206.68 | 0.19 | 0.72 | 3.96 | 183.29 | 32.86 | 1.04 | 159.95 |
| ***Percent < ^a^*** | 3.66 | 0.00 | 0.00 | 0.00 | 0.00 | 0.00 | 0.00 | 13.20 | 1.01 | 20.44 | 22.10 | 1.37 |
| ***Percent ><  ^b^*** | 78.53 | 0.38 | 64.39 | 0.00 | 20.00 | 92.94 | 95.69 | 86.80 | 80.81 | 58.01 | 71.82 | 19.18 |
| ***Percent > ^c^*** | 17.80 | 99.62 | 35.61 | 100.00 | 80.00 | 7.06 | 4.31 | 0.00 | 18.18 | 21.55 | 6.08 | 79.45 |

**Table 3B.** Biochemical features of included COVID-19 patients on admission, who had death outcome, COVID-19 referral treatment center, Shahid Sadoughi Hospital, Yazd, Iran (n=466).

|  | ***CPK*** | ***LDH*** | ***SGOT (AST)*** | ***SGPT (ALT)*** | ***Alkaline - P*** | ***Bilirubin D*** | ***Bilirubin T*** | ***Serum Albumin*** | ***Ferritin*** | ***Urea*** | ***Creatinine*** | ***FBS*** |
| --- | --- | --- | --- | --- | --- | --- | --- | --- | --- | --- | --- | --- |
| ***Counts*** | 157 | 157 | 157 | 157 | 157 | 157 | 157 | 157 | 157 | 157 | 157 | 157 |
| ***Average*** | 247.74 | 1300.46 | 68.99 | 62.47 | 254.46 | 0.36 | 1.04 | 3.70 | 635.14 | 72.93 | 1.68 | 193.80 |
| ***Percent < ^a^*** | 2.48 | 0.00 | 0.00 | 0.00 | 0.00 | 0.00 | 0.00 | 33.76 | 0.00 | 2.31 | 6.15 | 2.17 |
| ***Percent ><  ^b^*** | 66.94 | 0.00 | 45.56 | 0.59 | 15.43 | 88.46 | 87.82 | 66.24 | 17.14 | 30.00 | 61.54 | 9.42 |
| ***Percent > ^c^*** | 30.58 | 100.00 | 54.44 | 100.00 | 84.57 | 11.54 | 12.18 | 0.00 | 82.86 | 67.69 | 32.31 | 88.41 |

a: % of included COVID-19 patients showed the biochemical features lower than the normal range on admission.

b: % of included COVID-19 patients showed the biochemical feature between the normal range on admission.

C: % of included COVID-19 patients showed the biochemical features higher than the normal range on admission.

**Table 4A.** Hematological features of included COVID-19 patients on admission, who recovered on discharge, COVID-19 referral treatment center, Shahid Sadoughi Hospital, Yazd, Iran (n=466).

|  | ***Hematocrit*** | ***Hemoglobin*** | ***MCH*** | ***MCHC*** | ***MCV*** | ***MPV*** | ***PDW*** | ***PLT*** | ***R.B.C*** | ***W.B.C*** | ***PTT*** | ***PT*** | ***ESR_1 hr*** | ***Neutrophil*** | ***Lymphocyte*** | ***N/L*** | ***Het/Alb*** |
| --- | --- | --- | --- | --- | --- | --- | --- | --- | --- | --- | --- | --- | --- | --- | --- | --- | --- |
| ***Counts*** | 309 | 309 | 309 | 309 | 309 | 309 | 309 | 309 | 309 | 309 | 309 | 309 | 309 | 309 | 309 | 309 | 309 |
| ***Average*** | 36.69 | 12.07 | 28.59 | 32.78 | 87.16 | 10.27 | 13.16 | 205.24 | 4.23 | 8.28 | 36.25 | 12.91 | 51.46 | 77.76 | 17.28 | 8.15 | 9.16 |
| ***Percent < ^a^*** | 32.50 | 42.5 | 8.57 | 12.14 | 4.29 | 0.00 | 4.73 | 19.35 | 9.19 | 4.96 | 0.00 | 0.00 | 0.00 | 2.17 | 54.29 | 2.18 | 0.00 |
| ***Percent ><  ^b^*** | 67.50 | 57.7 | 91.07 | 87.14 | 93.57 | 89.42 | 90.91 | 80.29 | 90.81 | 80.50 | 92.91 | 0.00 | 24.80 | 26.35 | 43.57 | 18.91 | 100.00 |
| ***Percent > ^c^*** | 0.00 | 0.00 | 0.36 | 0.71 | 2.14 | 10.58 | 4.36 | 0.36 | 0.00 | 14.54 | 7.09 | 100.00 | 75.20 | 71.48 | 2.14 | 78.91 | 0.00 |

**Table 4B.** Hematological features of included COVID-19 patients on admission, who had death outcome, COVID-19 referral treatment center, Shahid Sadoughi Hospital, Yazd, Iran (n=466).

|  | ***Hematocrit*** | ***Hemoglobin*** | ***MCH*** | ***MCHC*** | ***MCV*** | ***MPV*** | ***PDW*** | ***PLT*** | ***R.B.C*** | ***W.B.C*** | ***PTT*** | ***PT*** | ***ESR_1 hr*** | ***Neutrophil*** | ***Lymphocyte*** | ***N/L*** | ***Het/Alb*** |
| --- | --- | --- | --- | --- | --- | --- | --- | --- | --- | --- | --- | --- | --- | --- | --- | --- | --- |
| ***Counts*** | 157 | 157 | 157 | 157 | 157 | 157 | 157 | 157 | 157 | 157 | 157 | 157 | 157 | 157 | 157 | 157 | 157 |
| ***Average*** | 37.57 | 12.22 | 28.84 | 32.49 | 88.76 | 10.71 | 14.63 | 175.19 | 314.83 | 12.52 | 41.18 | 14.79 | 59.96 | 85.43 | 9.94 | 20.56 | 10.34 |
| ***Percent < ^a^*** | 35.47 | 41 | 8.72 | 16.28 | 5.81 | 0.00 | 1.82 | 43.27 | 18.13 | 7.02 | 0.00 | 0.00 | 0.00 | 1.75 | 83.72 | 1.79 | 0.00 |
| ***Percent >< ^b^*** | 64.53 | 58.8 | 89.53 | 83.14 | 89.53 | 81.82 | 83.64 | 54.39 | 80.12 | 54.39 | 78.13 | 1.24 | 25.35 | 11.70 | 14.53 | 8.33 | 100.00 |
| ***Percent > ^c^*** | 0.00 | 0.2 | 1.74 | 0.58 | 4.65 | 18.18 | 14.55 | 2.34 | 1.75 | 38.60 | 21.88 | 98.76 | 74.65 | 86.55 | 1.74 | 89.88 | 0.00 |

a: % of included COVID-19 patients showed the biochemical features lower than the normal range on admission.

b: % of included COVID-19 patients showed the biochemical feature between the normal range on admission.

C: % of included COVID-19 patients showed the biochemical features higher than the normal range on admission.

**Table 5A.** Blood electrolyte features of included COVID-19 patients in admission, who recovered on their discharge, COVID-19 referral treatment center, Shahid Sadoughi Hospital, Yazd, Iran (n=466).

|  | ***Sodium*** | ***potassium*** | ***Calcium*** | ***Phosphorus*** | ***Magnesium*** | ***ph*** |
| --- | --- | --- | --- | --- | --- | --- |
| ***Counts*** | 309 | 309 | 309 | 309 | 309 | 309 |
| ***Average*** | 135.98 | 4.03 | 8.56 | 3.15 | 2.22 | 7.38 |
| ***Percent < ^a^*** | 27.47 | 7.22 | 90.32 | 38.61 | 1.25 | 24.69 |
| ***Percent >< ^b^*** | 71.79 | 89.53 | 9.27 | 55.70 | 63.75 | 45.68 |
| ***Percent > ^c^*** | 0.73 | 3.25 | 0.40 | 5.70 | 35.00 | 29.63 |

**Table 5B.** Blood electrolyte features of included COVID-19 patients on admission, who had death outcome, COVID-19 referral treatment center, Shahid Sadoughi Hospital, Yazd, Iran (n=466).

|  | ***Sodium*** | ***potassium*** | ***Calcium*** | ***Phosphorus*** | ***Magnesium*** | ***ph*** |
| --- | --- | --- | --- | --- | --- | --- |
| ***Counts*** | 157 | 157 | 157 | 157 | 157 | 157 |
| ***Average*** | 137.34 | 4.46 | 8.31 | 3.42 | 2.29 | 7.33 |
| ***Percent < ^a^*** | 26.63 | 6.51 | 94.63 | 38.89 | 2.80 | 41.55 |
| ***Percent >< ^b^*** | 69.23 | 73.96 | 5.37 | 50.00 | 52.34 | 36.62 |
| ***Percent > ^c^*** | 4.14 | 19.53 | 0.00 | 11.11 | 44.86 | 21.83 |

a: % of includud COVID-19 patients showed the biochemical features lower than the normal range on admission.

b: % of included COVID-19 patients showed the biochemical feature between the normal range on admission.

C: % of included COVID-19 patients showed the biochemical features higher than the normal range on admission.

**Table 6.** Comparison of biochemical parameters between two recovered and non-recovered groups of COVID-19 hospitalized patients, on admission (n=466).

|  |  | ***Mean + SE*** | |  |
| --- | --- | --- | --- | --- |
| ***Variable*** | ***Reference Value*** | ***Recovered (Mean + SE) (n=309)*** | ***Deceased (Mean + SE) (n=157)*** | ***p-value*** |
| ***CPK (U/L)*** | 24-200 | 222.33 + 31.06 | 284.79 + 26.647 | 0.578 |
| ***Troponin I (****-/+)* | Negative/positive | 2 (0.6%) | 3 (1.9%) | 0.211 |
| ***LDH(U/L)*** | 50-150 | 718.994 + 26.15 | 1283.61 + 151.55 | <0.001 |
| ***SGOT(U/L)*** | 6-40 | 42.99 + 2.077 | 71.99 + 11.15 | 0.001 |
| ***SGPT(U/L)*** | 5-56 | 40.77 +2.35 | 64.74 + 9.43 | 0.001 |
| ***Alkaline – P(U/L)*** | 42-128 | 209.58 + 8.97 | 245.16 + 20.10 | 0.02 |
| ***Bilirubin D(mg/dL)*** | 0-0.4 | 0.2027 + 0.00 | 0.361 + 0.064 | 0.001 |
| ***Bilirubin T(mg/dL)*** | 1-1.4 | 0.7578 + 0.05 | 1.01 + 0.10 | 0.012 |
| ***Albumin Serum(g/dL)*** | 3.5-5.5 | 3.952 + 0.029 | 3.683 + 0.05 | <0.001 |
| ***Urea(mg/dL)*** | 15-40 | 39.45 + 1.53 | 69.5 + 3.83 | <0.001 |
| ***Creatinine(mg/dL)*** | 0.7-1.4 | 1.139 + 0.07 | 1.623 + 0.114 | <0.001 |
| ***FBS(mg/dL)*** | 70-110 | 177.4 +1.97 | 190.78 + 6.29 | 0.011 |
| ***Ferritin(ng/mL)*** | 12-300 | 262.6744 + 8.78 | 377.351 + 15.99 | <0.001 |
| ***Sodium(mM)*** | 135-147 | 136.1 + 0.205 | 137.27 + 0.47 | 0.009 |
| ***Potassium(mM)*** | 3.5-5 | 4.042 + 0.024 | 4.49 + 0.066 | <0.001 |
| ***Calcium(mM)*** | 8.4-10.2 | 8.567 + 0.036 | 8.28 + 0.053 | <0.001 |
| ***Phosphorus(mM)*** | 3-4.5 | 3.1881 + 0.037 | 3.3799 + 0.112 | 0.044 |
| ***Magnesium(mM)*** | 1.5-2.3 | 2.2317 + 0.0158 | 2.281 + 0.0286 | 0.099 |

**Table 7.** Some laboratory findings of hospitalized patients on admission, with SGOT/SGPT higher than the normal (SGOT>40 & SGPT>56), and its association with sex and age (n=466).

| ***Variables*** | ***SGOT Ranges >40*** | | |  |  | ***Multivariate analysis for SGOT>40*** |  |
| --- | --- | --- | --- | --- | --- | --- | --- |
| ***Sex*** | ***Mean*** | ***p-value*** | ***SGOT >40(n=208)(%)*** | ***p-value*** | ***OR*** | ***95% CI*** | ***p-value*** |
| ***Male*** | 82.17 |  | 82 (56.6) |  | 2.014 | 1.354-2.998 | 0.001 |
| ***female*** | 85.94 | 0.811 | 126 (39.3) | 0.001 |  |  |  |
| ***Variables*** | ***SGPT Ranges >56*** | | |  |  | ***Multivariate analysis for SGPT>56*** |  |
| ***Sex*** | ***Mean*** | ***p-value*** | ***SGPT >56(n=108)(%)*** | ***p-value*** | ***OR*** | ***95% CI*** | ***p-value*** |
| ***Male*** | 100.59 |  | 59 (40.7) |  | 3.808 | 2.429-5.971 | <0.001 |
| ***female*** | 133.24 | 0.153 | 149 (15.3) | 0.001 |  |  |  |

**Table 8.** Some laboratory findings of hospitalized patients on admission, with SGOT/SGPT higher than the normal (SGOT>40 & SGPT>56), and its association with sex and age (n=466).

|  | ***AGOT=<40*** | ***SGOT>40*** |  |  | ***SGPT=<56*** | ***SGPT>56*** |  |
| --- | --- | --- | --- | --- | --- | --- | --- |
| ***Variables*** | ***Mean (n=258)*** | ***Mean (n=208)*** | ***p-value*** | ***Variables*** | ***Mean (n=358)*** | ***Mean (n=108)*** | ***p-value*** |
| ***Old*** | 43.85 | 49.59 | 0.003 | ***Old*** | 45.49 | 49.46 | 0.083 |
| ***CPK*** | 153.34 | 327.88 | <0.001 | ***CPK*** | 175.81 | 415 | <0.001 |
| ***LDH*** | 702.619 | 1165.485 | <0.001 | ***LDH*** | 823.724 | 1192.622 | 0.005 |
| ***SGOT*** | 27.21 | 84.46 | <0.001 | ***SGOT*** | 36.69 | 106.05 | <0.001 |
| ***SGPT*** | 26.24 | 76.88 | <0.001 | ***SGPT*** | 28.76 | 115.41 | <0.001 |
| ***Alkaline - P*** | 198.95 | 256.41 | 0.002 | ***Alkaline - P*** | 209.24 | 275.51 | 0.002 |
| ***Bilirubin D*** | 0.200 | 0.326 | 0.005 | ***Bilirubin D*** | 0.217 | 0.385 | 0.002 |
| ***Bilirubin T*** | 0.725 | 0.989 | 0.005 | ***Bilirubin T*** | 0.789 | 1.023 | 0.037 |
| ***W.B.C*** | 9.15 | 10.24 | 0.026 | ***Hematocrit*** | 36.661 | 38.267 | 0.016 |
| ***PT*** | 13.375 | 14.437 | 0.006 | ***Hemoglobin*** | 11.987 | 12.577 | 0.015 |
| ***Neutrophil*** | 7.275 | 8.416 | 0.033 | ***R.B.C*** | 4.222 | 4.378 | 0.041 |
| ***N/L*** | 11.030 | 14.395 | 0.023 | ***N/L*** | 11.459 | 16.086 | 0.008 |
| ***Ferritin*** | 269.913 | 340.254 | <0.001 | ***Het/Alb*** | 9.657 | 10.178 | 0.026 |
| ***Urea*** | 45.06 | 55.17 | 0.004 | ***Ferritin*** | 286.496 | 350.417 | 0.001 |
| ***Sodium*** | 135.86 | 137.28 | 0.001 | ***Urea*** | 46.6 | 59.44 | 0.002 |
| ***potassium*** | 4.114 | 4.29 | 0.003 | ***potassium*** | 4.135 | 4.382 | <0.001 |
| ***Magnesium*** | 2.214 | 2.292 | 0.006 | ***Magnesium*** | 2.231 | 2.305 | 0.03 |

**Table 9A.** The magnitude of CPK > 200 in hospitalized patients, on admission, and its association with sex.

| ***Variables*** | ***CPK Ranges >200*** | | |  |  | ***Multivariate analysis for CPK>200*** |  |
| --- | --- | --- | --- | --- | --- | --- | --- |
| ***Sex*** | ***Mean*** | ***p-value*** | ***CPK >200(n=223)(%)*** | ***p-value*** | ***OR*** | ***95% CI*** | ***p-value*** |
| ***Male*** | 609.42 |  | 78 (53.8) |  | 1.413 | 0.953-2.095 | 0.085 |
| ***female*** | 292.75 | <0.001 | 145 (45.2) | 0.085 |  |  |  |

**Table 9B.** Some laboratory findings of hospitalized patients, on admission, with different levels of CPK >< 200, and its association with sex and age.

| ***Variables*** | ***Mean(=<200)(n=243)*** | ***Mean(>200)(n=223)*** | ***p-value*** |
| --- | --- | --- | --- |
| ***LDH*** | 835.775 | 989.251 | 0.165 |
| ***SGOT*** | 42.55 | 63.89 | 0.008 |
| ***SGPT*** | 39.45 | 59.08 | 0.006 |
| ***Creatinine*** | 1.216 | 1.396 | 0.148 |
| ***Urea*** | 46.83 | 52.56 | 0.105 |
| ***PT*** | 13.371 | 14.369 | 0.01 |

**Table 10.** Some laboratory findings of hospitalized patients on admission, with serum different levels of CPK >< 200, and its association with deceased outcome.

|  | ***Recovered*** |  |  | ***Deceased*** |  |  |
| --- | --- | --- | --- | --- | --- | --- |
| ***Variables*** | ***Mean (CPK<200)(n=163)*** | ***Mean (CPK>200)(146)*** | ***p-value*** | ***Mean (CPK<200)(n=80)*** | ***Mean (CPK>200)(n=77)*** | ***p-value*** |
| ***Old*** | 38.84 | 40.87 | 0.334 | 59.60 | 59.25 | 0.909 |
| ***CPK*** | 67.22 | 395.49 | 0.00 | 85.23 | 418.73 | 0.00 |
| ***LDH*** | 632.64 | 815.40 | 0.00 | 1249.668 | 1318.883 | 0.82 |
| ***SGOT*** | 39.16 | 47.27 | 0.051 | 49.45 | 95.42 | 0.039 |
| ***SGPT*** | 36.28 | 45.78 | 0.044 | 45.91 | 84.31 | 0.041 |
| ***Urea*** | 31.94 | 47.82 | 0.00 | 77.15 | 61.56 | 0.041 |
| ***Creatinine*** | 0.944 | 1.357 | 0.004 | 1.769 | 1.47 | 0.193 |
| ***PT*** | 12.792 | 13.388 | 0.003 | 14.551 | 16.231 | 0.107 |

**Table 11.** The magnitude of some biochemical abnormalities in hospitalized patients, on admission, and its association with sex.

| ***Variables*** | ***Creatinine*** | | |  |  | ***Multivariate analysis for creatinine>1.4*** |  |
| --- | --- | --- | --- | --- | --- | --- | --- |
| ***Sex*** | ***Mean*** | ***p-value*** | ***Cases with Creatinine > 1.4(n=61)*** | ***p-value*** | ***OR*** | ***95% CI*** | ***p-value*** |
| ***Male*** | 4.345 |  | 35 (24.1%) |  | 4.696 | 2.054-6.650 | <0.001 |
| ***female*** | 2.327 | 0.02 | 26 (8.1%) | <0.001 |  |  |  |
|  | ***Urea*** | | |  |  | ***Multivariate analysis for Uremia>45*** | ***p-value*** |
| ***Sex*** | ***Mean*** | ***p-value*** | ***Cases with Urea > 45 (n=286)*** | ***p-value*** | ***OR*** | ***95% CI*** |  |
| ***Male*** | 80.26 |  | 115 (79.3%) |  | 3.053 | 1.955-4.767 | <0.001 |
| ***female*** | 58.71 | <0.001 | 171 (53.3%) | <0.001 |  |  |  |
|  | ***Calcium*** | | |  |  | ***Multivariate analysis for Hypocalcemia*** | ***p-value*** |
| ***Sex*** | ***Mean*** | ***p-value*** | ***Cases with Calcium < 8.4 (n=193)*** | ***p-value*** | ***OR*** | ***95% CI*** |  |
| ***Male*** | 7.788 |  | 73 (50.3%) |  | 1.690 | 1.137-2.512 | 0.010 |
| ***female*** | 8.002 | 0.002 | 120 (37.4%) | 0.006 |  |  |  |
|  | ***Potassium*** | | |  |  | ***Multivariate analysis for Hyperkalemia*** | ***p-value*** |
| ***Sex*** | ***Mean*** | ***p-value*** | ***Cases with potassium > 5 (n=51)*** | ***p-value*** | ***OR*** | ***95% CI*** |  |
| ***Male*** | 5.621 |  | 29 (20%) |  | 4.151 | 2.150-8.015 | <0.001 |
| ***female*** | 5.314 | <0.001 | 22 (6.9%) | <0.001 |  |  |  |

**Table 12A.** Some laboratory findings of admitted patients showing serum levels of Creatinine >1.4.

|  | ***Mean*** | | |
| --- | --- | --- | --- |
|  | ***Creatinine =< 1.4 (n=413)*** | ***Creatinine > 1.4(n=53)*** | ***p-value*** |
| ***CPK*** | 216.41 | 346.79 | 0.065 |
| ***LDH*** | 821.556 | 1592.336 | <0.001** |
| ***SGOT*** | 48.74 | 84.08 | 0.005** |
| ***SGPT*** | 45.87 | 71.98 | 0.020* |
| ***PLT*** | 199.86 | 149.64 | <0.001** |
| ***PT*** | 13.739 | 14.705 | 0.113 |
| ***Albumine Serum*** | 3.875 | 3.755 | 0.145 |
| ***Ferritin*** | 288.1186 | 404.1045 | <0.001** |
| ***Urea*** | 40.27 | 122.06 | <0.001** |
| ***Sodium*** | 136.25 | 138.43 | 0.001** |
| ***potassium*** | 4.097 | 4.932 | <0.001** |
| ***Calcium*** | 8.487 | 8.33 | 0.103 |
| ***Phosphorus*** | 3.1342 | 4.1755 | <0.001** |
| ***Magnesium*** | 2.2288 | 2.4068 | <0.001** |
| ***PLT*** | 201.54 | 145.03 | <0.001 |
| ***W.B.C*** | 10.281 | 5.44 | <0.001 |
| ***Neutrophils*** | 8.568949 | 4.517853 | <0.001 |
| ***Lymphocytes*** | 1.280406 | 0.557842 | <0.001 |
| ***Ferritin*** | 294.294 | 347.8923 | 0.03 |

**Table 12B.** Some laboratory findings of admitted patients showing serum levels of craetinine >1.4, and its association with age.

|  | ***Recovered*** | | | ***Deceased*** | | |
| --- | --- | --- | --- | --- | --- | --- |
| ***Variables*** | ***Mean*** | |  | ***Mean*** | |  |
|  | ***Creatinine< 1.4(n=297)*** | ***Creatinine > 1.4(n=12)*** | ***p-value*** | ***Creatinine<1.4(n=116)*** | ***Creatinine >1.4(n=41)*** | ***p-value*** |
| ***Old*** | 38.30 | 67.25 | 0.00 | 57.20 | 64.98 | 0.00 |
| ***CPK*** | 208 | 577 | 0.021 | 237.97 | 279.41 | 0.496 |
| ***LDH*** | 717.818 | 748.1 | 0.823 | 1087.162 | 1839.429 | 0.029 |
| ***LDH*** | 719.72 | 705.64 | 0.91 | 1074.42 | 1804.28 | 0.00 |
| ***SGOT*** | 43.28 | 37.75 | 0.56 | 62.01 | 96.84 | 0.02 |
| ***SGPT*** | 41.13 | 34.13 | 0.51 | 58.60 | 80.04 | 0.03 |
| ***Alkaline - P*** | 209.27 | 215.23 | 0.88 | 257.90 | 244.83 | 0.09 |
| ***Bilirubin D*** | 0.20 | 0.24 | 0.26 | 0.42 | 0.22 | 0.01 |
| ***Bilirubin T*** | 0.76 | 0.75 | 0.96 | 1.10 | 0.78 | 0.04 |
| ***Hematocrit*** | 36.94 | 32.01 | 0.00 | 38.38 | 36.08 | 0.07 |
| ***Hemoglobin*** | 12.16 | 10.25 | 0.00 | 12.50 | 11.63 | 0.34 |
| ***PLT*** | 0.2013 | 0.2383 | 0.012* | 182.4 | 151.69 | 0.08 |
| ***MPV*** | 10.29 | 10.56 | 0.31 | 10.62 | 10.89 | 0.02 |
| ***PDW*** | 13.23 | 13.90 | 0.31 | 14.22 | 15.50 | 0.00 |
| ***PLT*** | 205.97 | 171.63 | 0.12 | 185.21 | 147.42 | 0.01 |
| ***R.B.C*** | 4.26 | 3.82 | 0.01 | 4.41 | 4.06 | 0.47 |
| ***RDW-CV*** | 14.20 | 15.19 | 0.06 | 15.07 | 15.26 | 0.00 |
| ***RDW-SD*** | 46.89 | 50.51 | 0.02 | 48.92 | 51.31 | 0.00 |
| ***W.B.C*** | 8.59 | 11.43 | 0.00 | 11.12 | 12.12 | 0.00 |
| ***PT*** | 13.05 | 13.49 | 0.33 | 15.29 | 15.58 | 0.00 |
| ***ESR_1 hr*** | 51.74 | 59.55 | 0.24 | 60.04 | 59.82 | 0.01 |
| ***Neutrophil*** | 6.67 | 9.09 | 0.58 | 9.56 | 10.69 | 0.00 |
| ***Lymphocyte*** | 1.64 | 1.51 | 0.32 | 1.00 | 0.97 | 0.00 |
| ***N/L*** | 7.85 | 13.12 | 0.02 | 20.59 | 22.75 | 0.00 |
| ***Het/Alb*** | 9.47 | 8.20 | 0.00 | 10.72 | 10.01 | 0.00 |
| ***Albumine Serum*** | 3.95 | 3.96 | 0.95 | 3.69 | 3.66 | 0.00 |
| ***Ferritin*** | 257.35 | 360.15 | 0.01 | 364.20 | 410.07 | 0.00 |
| ***Urea*** | 36.15 | 99.81 | 0.00 | 47.97 | 123.11 | 0.00 |
| ***Creatinine*** | 1.00 | 3.69 | 0.00 | 1.04 | 3.07 | 0.01 |
| ***Sodium*** | 136.11 | 135.94 | 0.85 | 136.47 | 139.24 | 0.01 |
| ***potassium*** | 4.01 | 4.61 | 0.00 | 4.32 | 4.91 | 0.00 |
| ***Calcium*** | 8.58 | 8.31 | 0.09 | 8.25 | 8.34 | 0.00 |
| ***Phosphorus*** | 3.16 | 3.78 | 0.00 | 3.08 | 4.14 | 0.09 |
| ***Magnesium*** | 2.22 | 2.39 | 0.02 | 2.24 | 2.40 | 0.22 |
| ***pH*** | 7.35 | 7.31 | 0.02 | 7.34 | 7.31 | 0.11 |

**Table 13A.** Comparison of some hematological parameters between two recovered and non-recovered groups of COVID-19 hospitalized patients, on admission (n=466).

| ***Variables*** | ***Reference Value*** | ***Recovered (Mean + SE)*** | ***Deceased (Mean + SE)*** | ***p-value*** |
| --- | --- | --- | --- | --- |
| ***W.B.C(×10^3^/µL)*** | 3.5-11 | 8.753 + 0.217 | 11.407 + 0.557 | <0.001 |
| ***Neutrophil (×10^3^/µL)*** | 1.3-8 | 6.967 + 0.352 | 10.148 + 0.652 | <0.001 |
| ***Lymphocyte (×10^3^/µL)*** | 1-4.8 | 1.369 + 0.135 | 0.823 + 0.088 | <0.001 |
| ***N/L*** | 1-3 | 8.124 + 0.506 | 21.206 + 1.75 | <0.001 |
| ***Het/Alb*** | <7 | 9.405 + 0.098 | 10.51 + 0.21 | <0.001 |
| ***ESR_1 hr(mm/h)*** | 0-29 | 52.143 + 1.484 | 59.977 + 2.370 | 0.004 |
| ***PLT(×10^9^/L)*** | 140-450 | 204.19 + 4.90 | 174.38 + 7.70 | 0.001 |
| ***PTT*** | 18-45 | 48.06 + 1.73 | 50.39 + 2.435 | 0.437 |
| ***PT(s)*** | 10-13 | 13.074 + 0.099 | 15.375 + 0.520 | <0.001 |
| ***MPV(fL)*** | 7.2-11.7 | 10.3059 + 0.059 | 10.69 + 0.091 | <0.001 |
| ***PDW%*** | 10-17.9 | 13.2669 + 0.143 | 14.587 + 0.252 | <0.001 |
| ***Ferritin(ng/mL)*** | 12-300 | 262.6744 + 8.78 | 377.351 + 15.99 | <0.001 |
| ***CRP (-)*** | % of total (n=50) | 90% | 10% |  |
| ***CRP (+1)*** | % of total (n=56) | 66.1% | 33.9% |  |
| ***CRP (+2)*** | % of total (n=236) | 60.6% | 39.4% |  |
| ***CRP (+3)*** | % of total (n=124) | 67.7% | 32.3% | 0.001 |

**Table 13B.** Comparison of some hematological parameters between two recovered and non-recovered groups of COVID-19 hospitalized patients, on admission (n=466).

|  |  | ***Mean + SE*** | | |
| --- | --- | --- | --- | --- |
| ***Variables*** | ***Reference Value*** | ***Recovered (Mean + SE)*** | ***Deceased (Mean + SE)*** | ***p-value*** |
| ***Hematocrit (%)*** | 35-62 | 36.684 + 0.32 | 37.72 + 0.54 | 0.081 |
| ***Hemoglobin(g/dL)*** | 12-17.5 | 12.059 + 0.117 | 12.25 + 0.20 | 0.383 |
| ***MCH(pg/cell)*** | 25-35 | 28.7188 + 0.157 | 28.54 + 0.232 | 0.539 |
| ***MCHC(g/dL)*** | 31-36 | 32.7666 + 0.086 | 34.43 + 0.142 | 0.038 |
| ***MCV(fL)*** | 76-101 | 87.5989 + 0.41 | 87.92 + 0.592 | 0.601 |
| ***R.B.C(× 10^12^/L)*** | 3.5-5.5 | 4.2329 + 0.035 | 4.30 + 0.067 | 0.277 |
| ***RDW-CV(%)*** | 11.5-13.5 | 14.25 + 0.115 | 15.123 + 0.198 | <0.001 |
| ***RDW-SD(%)*** | 39-46 | 47.08 + 0.3375 | 49.60 + 0.580 | <0.001 |
| ***pH*** | 7.31-7.41 | 7.3504 + 0.003 | 7.330 + 0.011 | 0.042 |
| ***pCO2(mmHg)*** | 41-51 | 42.339 + 0.322 | 44.84 + 1.252 | 0.013 |
| ***HCO3(mM)*** | 18-23 | 23.246 + 0.162 | 23.12 + 0.511 | 0.77 |
| ***pO2(mmHg)*** | 30-40 | 43.602 + 0.553 | 44.92 + 2.298 | 0.466 |
| ***O2sat(%)*** | 75 | 67.707 + 0.622 | 65.844 + 1.755 | 0.223 |

**Table 14.** The magnitude and laboratory findings of some hematological abnormalities in hospitalized patients on admission and the association with sex.

| ***Variables*** | ***Lymphocytes <1*** | | |  |  | ***Multivariate analysis for Lymphopenia*** |  |
| --- | --- | --- | --- | --- | --- | --- | --- |
| ***Sex*** | ***Mean*** | ***p-value*** | ***Cases with Lymphopenia (n=244) (%)*** | ***p-value*** | ***OR*** | ***95% CI*** | ***p-value*** |
| ***Male*** | 0.520 |  | 111 (76.6) |  | 4.615 | 2.961-7.192 | <0.001 |
| ***female*** | 0.612 | 0.004 | 133 (41.4) | <0.001 |  |  |  |
|  | ***Neutrophils >8*** | | |  |  | ***Multivariate analysis for Neutrophilia*** |  |
|  | ***Mean*** | ***p-value*** | ***Cases with Neutrophilia (n=178)(%)*** | ***p-value*** | ***OR*** | ***95% CI*** | ***p-value*** |
| ***Male*** | 12.760 |  | 77 (53.1) |  | 2.467 | 1.649-3.688 | <0.001 |
| ***female*** | 12.361 | 0.605 | 101 (31.5) | <0.001 |  |  |  |
|  | ***PLT<140*** | | |  |  | ***Univariate analysis for Thrombocytopenia*** |  |
|  | ***Mean*** | ***p-value*** | ***Cases with Thrombocytopenia (n=132) (%)*** | ***p-value*** | ***OR*** | ***95% CI*** | ***p-value*** |
| ***Male*** | 96.78 |  | 66(45.5) |  | 3.173 | 2.070-4.863 | <0.001 |
| ***female*** | 98.78 | 0.743 | 66(20.6) | <0.001 |  |  |  |
|  | ***RBC<3.5*** | | |  |  | ***Univariate analysis for Anemia*** |  |
|  | ***Mean*** | ***p-value*** | ***Cases with Anemia (n=95)(%)*** | ***p-value*** | ***OR*** | ***95% CI*** | ***p-value*** |
| ***Male*** | 2.962 |  | 32(22.1) |  | 3.081 | 1.768-5.378 | <0.001 |
| ***female*** | 3.049 | 0.01 | 27 (8.4) | <0.001 |  |  |  |

**Table 15A.** Some laboratory findings of admitted patients with lymphocyte counts ><1.

| ***Variables*** | ***Mean (Lymphocytes Range <1)*** | ***Mean (Lymphocytes Range >=1)*** | ***p-value*** |
| --- | --- | --- | --- |
| ***Old*** | 50.94 | 36.77 | <0.001 |
| ***CPK*** | 261.82 | 166.18 | 0.047 |
| ***LDH*** | 997.762 | 720.844 | 0.019 |
| ***PDW*** | 14.069 | 12.9514 | <0.001 |
| ***MPV*** | 10.5703 | 10.1522 | <0.001 |
| ***W.B.C*** | 10.75 | 7.27 | <0.001 |
| ***Neutrophil*** | 86.897 | 67.537 | <0.001 |
| ***Lymphocyte*** | 8.409 | 27.439 | <0.001 |
| ***N/L ratio*** | 17.037 | 2.945 | <0.001 |
| ***Het/Alb*** | 10.005 | 9.295 | 0.001 |
| ***Ferritin*** | 324.8671 | 251.1923 | <0.001 |
| ***Urea*** | 55.06 | 37.89 | <0.001 |
| ***Creatinine*** | 1.406 | 1.082 | 0.015 |
| ***potassium*** | 4.282 | 4.001 | <0.001 |
| ***Calcium*** | 8.409 | 8.597 | 0.004 |

**Table 15B.** Some laboratory findings of admitted patients with lymphopenia (<1) and its association with deceased outcome.

|  | ***Recovered*** | | | ***Deceased*** | | |
| --- | --- | --- | --- | --- | --- | --- |
| ***Variables*** | ***Mean (Lymphocytes Range <1)*** | ***Mean (Lymphocytes Range >=1)*** | ***p-value*** | ***Mean (Lymphocytes Range <1)*** | ***Mean (Lymphocytes Range >=1)*** | ***p-value*** |
| ***Old*** | 42.61 + 1.42 | 36.08 + 1.50 | 0.002 | 61.35 + 1.42 | 42.5 +7.29 | <0.001 |
| ***W.B.C*** | 9.86 + 0.3 | 7.26 + 0.3 | <0.001 | 11.87 + 0.6 | 7.29 + 1.2 | 0.013 |
| ***Neutrophil*** | 8.468 + 0.351 | 5.00 + 0.227 | <0.001 | 10.744 + 0.65 | 4.89 + 0.378 | <0.001 |
| ***N/L*** | 12.009 + 0.8 | 2.984 + 0.1 | <0.001 | 23.32 + 1.9 | 2.63 + 0.3 | <0.001 |
| ***Lymphocyte*** | 0.814 + 0.043 | 1.95 + 0.184 | <0.001 | 0.684 + 0.335 | 2.053 + 0.16 | <0.001 |
| ***Het/Alb*** | 9.538 + 0.1 | 9.228 + 0.1 | 0.118 | 10.587 + 0.2 | 9.855 + 0.583 | 0.293 |
| ***Albumine Serum*** | 3.897 + 0.0 | 4.025 + 0.0 | 0.028 | 3.666 + 0.1 | 3.835 + 0.2 | 0.307 |
| ***R.B.C*** | 4.245 + 0.0 | 4.217 + 0.1 | 0.693 | 4.3055 + 0.1 | 4.325 + 0.1 | 0.93 |
| ***Hemoglobin*** | 12.021 + 0.2 | 12.11 + 0.2 | 0.709 | 12.268 + 0.2 | 12.094 + 0.6 | 0.795 |
| ***RDW-CV*** | 14.175 + 0.1 | 14.348 + 0.2 | 0.459 | 15.191 + 0.2 | 14.531 + 0.9 | 0.316 |
| ***MCH*** | 28.7571 + 0.2 | 28.668 + 0.3 | 0.78 | 28.5979 + 0.2 | 28.115 + 0.3 | 0.532 |
| ***PLT*** | 201.91 + 6.3 | 207.21 + 7.8 | 0.594 | 175.2 + 8.3 | 167.13 + 20.0 | 0.752 |
| ***PDW*** | 13.5876 + 0.2 | 12.8425 + 0.2 | 0.01 | 14.67 + 0.3 | 13.8569 + 0.6 | 0.332 |
| ***ESR_1 hr*** | 52.381 + 2.1 | 51.829 + 2.1 | 0.854 | 61.184 + 2.5 | 49.337 + 6.8 | 0.131 |
| ***Ferritin*** | 278.15 + 12.9 | 242.2 + 11 | 0.043 | 383.19 + 17.3 | 325.94 + 34.6 | 0.28 |
| ***PT*** | 13.261 + 0.2 | 12.826 + 0.1 | 0.03 | 15.161 + 0.5 | 17.259 + 3 | 0.224 |
|  | ***% of Cases recovered*** | |  | ***% of Cases deceased*** | |  |
| ***CRP (-)*** | 10.8 | 19.5 |  | 3.5 | 0.0 |  |
| ***CRP (+1)*** | 9.7 | 15.0 |  | 9.9 | 31.3 |  |
| ***CRP (+2)*** | 51.7 | 39.1 |  | 63.1 | 25.0 |  |
| ***CRP (+3)*** | 27.8 | 26.3 | 0.037 | 23.4 | 43.8 | 0.008 |
| ***% of Total cases*** | 57 | 43 |  | 89.8 | 10.2 | <0.001 |

**Table 16A.** The magnitude and laboratory findings of hemoglobin <12 in hospitalized patients on admission and the association with sex.

| ***Variables*** | ***Hemoglobin*** | | |  |  | ***Multivariate analysis for Lymphopenia*** |  |
| --- | --- | --- | --- | --- | --- | --- | --- |
| ***Sex*** | ***Mean*** | ***p-value*** | ***Cases with Hemoglobinemia (%)*** | ***p-value*** | ***OR*** | ***95% CI*** | ***p-value*** |
| ***Male*** | 9.334 |  | 30 (42.3) |  | 1.210 | 0.809-1.795 | 0.359 |
| ***female*** | 10.418 | 0.001 | 105 (44.1) | 0.782 |  |  |  |

**Table 16B.** Some laboratory findings of admitted patients with serum levels of Hemoglobin ><12.

|  | ***Mean*** |  |  |
| --- | --- | --- | --- |
| ***Variables*** | ***Hb <12 (n=201)*** | ***Hb >12 (n=265)*** | ***p-value*** |
| ***Bilirubin T*** | 0.9671 | 0.7488 | 0.022 |
| ***Hematocrit*** | 31.85 | 40.964 | <0.001 |
| ***Hemoglobin*** | 10.145 | 13.624 | <0.001 |
| ***MCH*** | 27.8424 | 29.2828 | <0.001 |
| ***MCHC*** | 31.8251 | 33.2863 | <0.001 |
| ***R.B.C*** | 3.789 | 4.6138 | <0.001 |
| ***RDW-CV*** | 15.389 | 13.904 | <0.001 |
| ***RDW-SD*** | 49.655 | 46.622 | <0.001 |
| ***PT*** | 14.437 | 13.403 | 0.008 |
| ***PLT*** | 189.77 | 197.47 | 0.366 |
| ***W.B.C*** | 9.73 | 9.584 | 0.766 |
| ***Neutrophil*** | 7.964 | 8.096 | 0.777 |
| ***Lymphocyte*** | 1.335 | 1.073 | 0.024 |
| ***N/L*** | 11.626 | 13.218 | 0.284 |
| ***Het/Alb*** | 8.768 | 10.544 | <0.001 |
| ***Albumine Serum*** | 3.721 | 3.969 | <0.001 |
| ***Ferritin*** | 292.606 | 307.912 | 0.362 |
| ***Alkaline - P*** | 242.26 | 211.2 | 0.049 |
| ***Bilirubin D*** | 0.315 | 0.212 | 0.023 |
| ***Urea*** | 54.62 | 45.74 | 0.013 |
| ***Creatinine*** | 1.513 | 1.142 | 0.003 |
| ***Calcium*** | 8.347 | 8.562 | <0.001 |
| ***pco2*** | 41.785 | 44.24 | 0.01 |
| ***HCO3*** | 22.569 | 23.685 | 0.006 |

**Table 17.** Some laboratory findings of admitted patients with hemoglobin ><12 and its association with deceased outcome.

|  | ***Recovered*** | | | ***Deceased*** | | |
| --- | --- | --- | --- | --- | --- | --- |
| ***Variables*** | ***Mean (Hb<12)(n=135)*** | ***Mean (Hb >12)(n=174)*** | ***p-value*** | ***Mean (Hb<12)(n=66)*** | ***Mean (Hb>12)(n=91)*** | ***p-value*** |
| ***Old*** | 40.19 | 39.5 | 0.746 | 60.38 | 58.74 | 0.598 |
| ***Bilirubin D*** | 0.219 | 0.1901 | 0.074 | 0.5115 | 0.2525 | 0.046 |
| ***Bilirubin T*** | 0.8455 | 0.6898 | 0.115 | 1.2158 | 0.8618 | 0.085 |
| ***Hematocrit*** | 32.118 | 40.226 | <0.001 | 31.302 | 42.376 | <0.001 |
| ***Hemoglobin*** | 10.299 | 13.425 | <0.001 | 9.829 | 14.006 | <0.001 |
| ***MCH*** | 27.8541 | 29.3896** | <0.001 | 27.8183** | 29.0785 | 0.007 |
| ***MCHC*** | 31.9444 | 33.4045** | <0.001 | 31.5812** | 33.0601 | <0.001 |
| ***MCV*** | 87.1374 | 87.957 | 0.322 | 87.993 | 87.9575 | 0.976 |
| ***MPV*** | 10.2347 | 10.361 | 0.291 | 10.7438 | 10.6577 | 0.645 |
| ***PDW*** | 13.3202 | 13.2255 | 0.745 | 14.9686 | 14.3104 | 0.2 |
| ***PLT*** | 207.72 | 201.45 | 0.528 | 153.06 | 189.84 | 0.018 |
| ***R.B.C*** | 3.8737 | 4.5117 | <0.001 | 3.6158 | 4.8092 | <0.001 |
| ***RDW-CV*** | 14.939 | 13.715 | <0.001 | 16.308 | 14.265 | <0.001 |
| ***RDW-SD*** | 48.383 | 46.069 | 0.001 | 52.255 | 47.68 | <0.001 |
| ***PT*** | 13.303 | 12.895 | 0.042 | 16.755 | 14.374 | 0.023 |
| ***PLT*** | 207.72 | 201.45 | 0.528 | 153.06 | 189.84 | 0.018 |
| ***W.B.C*** | 8.95 | 8.58 | 0.388 | 11.29 | 11.49 | 0.863 |
| ***Neutrophil*** | 6.980949 | 6.955823 | 0.951 | 9.973734 | 10.274789 | 0.776 |
| ***Lymphocyte*** | 1.556578 | 1.224896 | 0.033 | 0.881228 | 0.781999 | 0.489 |
| ***N/L*** | 7.570 | 8.554 | 0.336 | 19.922 | 22.138 | 0.534 |
| ***Het/Alb*** | 8.585 | 10.041 | <0.001 | 9.143 | 11.506 | <0.001 |
| ***Albumine Serum*** | 3.818 | 4.056 | <0.001 | 3.521 | 3.801 | 0.005 |
| ***Ferritin*** | 261.914 | 263.264 | 0.939 | 355.385 | 393.282 | 0.243 |
| ***Alkaline - P*** | 209.39 | 209.73 | 0.985 | 309.5 | 214.02 | 0.019 |
| ***Bilirubin D*** | 0.219 | 0.1901 | 0.074 | 0.5115 | 0.2525 | 0.046 |
| ***Urea*** | 42.24 | 37.28 | 0.108 | 79.95 | 61.92 | 0.02 |
| ***Creatinine*** | 1.291 | 1.021 | 0.063 | 1.968 | 1.372 | 0.01 |
| ***Calcium*** | 8.483 | 8.632 | 0.039 | 8.068 | 8.429 | 0.001 |
| ***pco2*** | 42.41 | 42.29 | 0.856 | 40.51 | 47.97 | 0.003 |
| ***HCO3*** | 22.967 | 23.462 | 0.13 | 21.753 | 24.112 | 0.022 |

**Table 18.** Correlations between biochemical parameters in COVID-19 patients with recovered or deceased outcomes.

|  | ***Recovered*** |  | ***Deceased*** |  |
| --- | --- | --- | --- | --- |
| ***Correlated Parameter*** | ***r*** | ***p-value*** | ***r*** | ***p-value*** |
| ***CPK-Creatinine*** | 0.370** | <0.001 | 0.002 | 0.978 |
| ***CPK-Urea*** | 0.354** | <0.001 | -0.126 | 0.116 |
| ***CPK-SGOT*** | 0.231** | <0.001 | 0.240** | 0.002 |
| ***CPK-SGPT*** | 0.264** | <0.001 | 0.240** | 0.002 |
| ***CPK-LDH*** | 0.351** | <0.001 | 0.214** | 0.007 |
| ***Bilirubin*** | ***Recovered*** | | ***Deceased*** | |
| ***Parameter*** | ***r*** | ***p-value*** | ***r*** | ***p-value*** |
| ***BiliD-AlkP*** | 0.232** | <0.001 | 0.755** | <0.001 |
| ***BiliD-PLT*** | -0.120* | 0.035 | -0.214** | 0.007 |
| ***BiliD-PT*** | 0.250** | <0.001 | 0.319** | 0.001 |

*: Correlation is significant at the 0.05 level (2-tailed).

**: Correlation is significant at the 0.01 level (2-tailed).

**Table 19A.** Multivariate analysis of biochemical factors and inflammatory markers that might be associated with COVID-19 risk of death.

|  |  | ***Multivariate analysis of risk factors, 95% CI*** | |  |
| --- | --- | --- | --- | --- |
| ***Deceased outcome*** | ***OR*** | ***Lower*** | ***Upper*** | ***p-value*** |
| ***BilirubinD*** | 7.347 | 2.134 | 25.297 | 0.002 |
| ***Alkaline-p (>128)*** | 1.518 | 0.842 | 2.739 | 0.166 |
| ***Urea (>40)*** | 3.410 | 2.187 | 5.316 | <0.001 |
| ***Creatinine*** | 1.460 | 1.151 | 1.851 | 0.002 |
| ***Potassium*** | 3.506** | 2.413 | 5.094 | <0.001 |
| ***Calcium*** | 0.493** | 0.357 | 0.679 | <0.001 |
| ***Phosphorus*** | 0.951 | 0.763 | 1.186 | 0.655 |
| ***Magnesium*** | 1.687 | 0.906 | 3.153 | 0.01 |

**Table 19B.** Multivariate analysis of inflammatory markers may be associated with COVID-19 risk of death.

|  |  | ***Multivariate analysis of risk factors, 95% CI*** | |  |
| --- | --- | --- | --- | --- |
| ***Deceased outcome*** | ***OR*** | ***Lower*** | ***Upper*** | ***p-value*** |
| ***D-dimer (>200)*** | 1.337 | 0.905 | 1.976 | 0.144 |
| ***N/L ratio (>9)*** | 5.59** | 3.682 | 8.485 | <0.001 |
| ***Het/Alb*** | 1.295** | 1.169 | 1.433 | <0.001 |
| ***CRP (+3)*** | 4.622** | 1.574 | 13.567 | <0.001 |
| ***CRP (+2)*** | 5.853** | 2.241 | 15.288 | 0.004 |
| ***CRP (+1)*** | 4.714** | 1.580 | 11.623 | 0.003 |
| ***Albumin*** | 0.532** | 0.36 | 0.786 | 0.002 |
| ***Ferritin (>300)*** | 10.981** | 4.35 | 27.719 | <0.001 |

**Table 19C.** Multivariate analysis of hematological factors may be associated with COVID-19 risk of death.

|  |  | ***Multivariate analysis of risk factors, 95% CI*** | |  |
| --- | --- | --- | --- | --- |
| ***Deceased outcome*** | ***OR*** | ***Lower*** | ***Upper*** | ***p-value*** |
| ***Neutrophilia(>8)*** | 3.813** | 2.086 | 6.972 | <0.001 |
| ***Lymphocytopenia (<1)*** | 6.659** | 3.789 | 11.705 | <0.001 |
| ***RBC*** | 0.748 | 0.455 | 1.266 | 0.301 |
| ***BilirubinT*** | 1.333* | 1.038 | 1.711 | 0.025 |
| ***Hematocrit*** | 1.311** | 1.053 | 1.829 | 0.001 |
| ***Hemoglobin*** | 0.585** | 0.413 | 0.828 | 0.003 |
| ***RDW-CV*** | 1.26** | 1.142 | 1.392 | <0.001 |
| ***PLT (<140)*** | 3.062** | 2.0130 | 4.657 | <0.001 |
| ***PT (>13)*** | 4.463** | 2.648 | 7.522 | <0.001 |

**Table 20A.** Multivariate analysis for the association of age with lymphopenia and neutrophilia in COVID-19 admitted patients (n=466).

| ***Variables*** | ***Lymphocytes*** |  |  |  |  | ***Univariate analysis of risk factors*** | ***p-value*** |
| --- | --- | --- | --- | --- | --- | --- | --- |
| ***Age*** | ***Mean*** | ***p-value*** | ***Lymphopenia (n=317)(%)*** | ***p-value*** | ***OR*** | ***(Lymphopenia) 95% CI*** |  |
| ***18-40(n=232)*** | 1.433976 |  | 120 (51.7) |  |  |  | <0.001 |
| ***41-60(n=87)*** | 0.979 |  | 70 (80.5) |  | 3.843 | 2.132-6.927 | <0.001 |
| ***61-100 (n=147)*** | 0.872 | <0.001 | 127 (86.4) | <0.001 | 5.927 | 3.463-10.142 | <0.001 |

| ***Variables*** | ***Neutrophils*** |  |  |  |  | ***Univariate analysis of risk factors*** | ***p-value*** |
| --- | --- | --- | --- | --- | --- | --- | --- |
| ***Age*** | ***Mean*** | ***p-value*** | ***Neutrophilia (365)(%)*** | ***p-value*** | ***OR*** | ***(Neutrophilia) 95% CI*** |  |
| ***18-40(n=232)*** | 6.604 |  | 158 (68.1) |  |  |  | <0.001 |
| ***41-60(n=87)*** | 8.857 |  | 76 (87.4) |  | 3.045 | 1.561-5.94 | 0.001 |
| ***61-100 (n=147)*** | 10.491 | <0.001 | 134 (91.2) | <0.001 | 5.022 | 2.669-9.447 | <0.001 |

**Table 20B.** Multivariate analysis for the association of age with lymphopenia and neutrophilia in COVID-19 admitted patients (n=466).

| ***Lymphopenia*** | | | | | |
| --- | --- | --- | --- | --- | --- |
| ***Sex*** | ***Age (Years)*** | ***OR*** | ***Lower*** | ***Upper*** | ***p-value*** |
| ***Male patients*** | 18-40 |  |  |  | 0.025 |
|  | 41-60 | 3.90 | 1.184 | 12.85 | 0.004 |
|  | 61-100 | 7.00 | 2.164 | 22.642 | 0.001 |
|  |  |  |  |  |  |
| ***Female patients*** | 18-40 |  |  |  | 0.007 |
|  | 41-60 | 2.765 | 1.319 | 5.792 | <0.001 |
|  | 61-100 | 3.994 | 2.096 | 7.61 | <0.001 |

| ***Neutrophilia*** | | | | | |
| --- | --- | --- | --- | --- | --- |
| ***Sex*** | ***Age (Years)*** | ***OR*** | ***Lower*** | ***Upper*** | ***p-value*** |
| ***Male patients (n=145)*** | 18-40 |  |  |  | 0.031 |
|  | 41-60 | 4.00 | 1.136 | 14.085 | 0.004 |
|  | 61-100 | 5.833 | 1.775 | 19.169 | 0.01 |
|  |  |  |  |  |  |
| ***Female patients(n=321)*** | 18-40 |  |  |  | 0.044 |
|  | 41-60 | 2.429 | 1.026 | 5.749 | 0.001 |
|  | 61-100 | 4.441 | 1.932 | 10.208 | <0.001 |
